# Suicide risk factors in nursing students: Depressive symptoms, academic burnout and academic efficacy after the pandemic

**DOI:** 10.1371/journal.pmen.0000734

**Published:** 2026-11-10

**Authors:** Rodrigo-Alejandro Ardiles-Irarrázabal, Pablo Alejandro Pérez-Díaz, Juan-Carlos Pérez-González, Yenny Valdivia-Rojas, Miguel Valencia-Contrera

**Affiliations:** 1 Departamento de Enfermería, Universidad de Antofagasta, Antofagasta, Chile; 2 Faculty of Psychology, San Sebastian University, Sede Valdivia, Chile; 3 Faculty of Education, Universidad Nacional de Educación a Distancia (UNED), Madrid, Spain; 4 Faculty of Nursing, University of Concepción, Concepción, Chile; Affiliated Guangji Hospital of Soochow University, CHINA

## Abstract

The present study analyze the prevalence and factors associated with suicide risk in nursing students in the post-pandemic context, considering academic burnout, academic engagement, and symptoms of depression, anxiety, and stress as predictors. through a cross-sectional and correlational study conducted in a public university in Northern Chile. with a168 nursing undergraduates’ sample. Validated instruments were administered, including the Beck Hopelessness Scale, the Utrecht Work Engagement Scale, the Maslach Burnout Inventory–Student Survey, and the DASS-21. Descriptive analyses, Pearson correlations, and multiple linear regression were conducted. A total of 11.31% of students met the predefined BHS threshold for elevated suicide-related risk. In the same sample, symptoms of depression, anxiety, and stress showed high prevalence rates. Suicide risk was negatively associated with vigor and dedication, while positively associated with low academic efficacy and depression. The regression model explained 54% of the variance in suicide risk (*Adj.R²* = 0.54; *p* < 0.001), with Depression (*β* = 4.04), Vigor (*β* = 0.63), Academic Efficacy (*β* = 0.88), and the interaction between Depression and Vigor (*β* = -0.69) being significant predictors in the model. Suicide risk in nursing students transcends traditional clinical factors, as it is shaped by the interaction between personal resources and mental health symptoms. These findings underscore the need for prevention and psychosocial support programs in nursing schools.

## Introduction

Suicide risk is a significant public health issue, particularly among university students. Nursing students may experience factors associated with elevated suicide-related risk because of the academic, emotional, and clinical demands of their training. However, suicide risk is a complex and dynamic construct that can vary over time according to psychological symptoms, life events, interpersonal circumstances, and contextual stressors. Accordingly, in this study, suicide risk is understood as an increased likelihood of suicide-related thoughts or behaviors associated with established psychological risk indicators, rather than as a fixed or diagnostic condition [1,2]. In this context, it is a priority to identify the factors associated with suicide risk in relation to the academic and personal demands placed on these students.

The COVID-19 pandemic reported a 25% increase in the global prevalence of anxiety and depression [3]. These problems had a significant impact on students in general and on nursing students in particular [4–6]. After lockdown, the return to face-to-face learning was associated with significant socio-emotional repercussions, such as anxiety, depression, burnout, and difficulties in conflict resolution [7,8]. In the post-pandemic period, there is still a need to characterize the mental health of this group.

Before the pandemic, university students in Chile already had high prevalences of anxiety and depression [9]. Health students had even higher prevalence rates, with greater academic stress observed in this population compared to other disciplines [10], and nursing students were among the most susceptible [6,11], being associated with various mental health disorders compared to the general university population [12,13]. A recent umbrella review synthesis of meta-analytic evidence confirmed this trend, reporting an overall prevalence of 27% of mental health disorders in nursing students [14].

Suicidal behavior exhibits a gender paradox: men have higher rates of suicide, while women make more suicide attempts [15,16]. Between 2007 and 2022, attempts among women aged 15–24 increased by 87% [13], a finding particularly relevant in the United States, where most of the population is female [15,16]. Following social isolation, the main psycho-emotional problems reported included subjective distress, burnout, anxiety, and depression [14,17,18]. Therefore, it is essential to identify the factors associated with suicide risk among nursing students in the presented scenario.

An umbrella review synthesis showed high prevalence rates of symptoms of depression, anxiety, and stress among nursing students, estimated at 29%, 29%, and 27%, respectively [14]. In Chile, before the pandemic, these students already had higher levels of trait anxiety than any other health professionals [19,20]. During the pandemic, more than 80% reported suffering from mild anxiety, depression, or stress [21]. Furthermore, nursing education combines substantial academic demands with progressively intensive clinical training, requiring students to interact directly with patients and families while learning to manage pain, suffering, illness, and death. These emotionally demanding experiences may distinguish nursing students from students in disciplines with less sustained clinical exposure and may contribute to additional psychological strain during training [22]. In the post-pandemic scenario, it is necessary to assess whether these variables continue to play a role in suicide risk.

Chronic stress in the university setting manifests as academic burnout syndrome (ABS), associated with deterioration in health and performance [23–27]. The review reported a 32% prevalence of burnout in the United States [14]. However, in Chile, 100% of cases were reported in the pre-pandemic stage [28]. During the pandemic, nursing students also experienced substantial stress and a need for institutional strategies aimed at strengthening coping and resilience, with student-led support groups showing potential benefits for stress management [29]. During the pandemic, academic burnout affected 75% of students across its three dimensions (emotional exhaustion, cynicism, and academic efficacy), and strong positive correlations were observed between academic burnout syndrome and suicide risk [6]. In the post-lockdown period, the prevalence of moderate academic burnout syndrome was 53% [30].

The proportion of nursing students identified as presenting elevated suicide-related risk varies substantially across studies, partly because different instruments and operational definitions have been used. Reported estimates range from 8% to 53.3% in pre-pandemic studies [13,31,32], approximately 10.5% during the pandemic [6], and between 17.5% and 19.3% following lockdown [8,33]. These figures should therefore be interpreted as screening-based estimates of elevated risk rather than as the prevalence of a stable clinical condition.

Academic engagement has been described as a protective factor against psychological maladjustment in university students. During the pandemic, it also acted as a buffer against stress-related symptoms and adverse mental health outcomes [34,35]. This raises the question of its role in the post-pandemic period, as its interaction with academic burnout syndrome and depression, anxiety, and stress to explain suicide risk remains largely unexplored.

The purpose of this study was to identify factors associated with suicide risk in nursing students at a public university in the post-pandemic context. The research examines potential risk and protective factors, including depression, anxiety, stress, academic burnout, and academic engagement, to promote early detection, guide future research, and inform the design of preventive strategies for this healthcare group.

The hypothesis proposed for the present study was that suicide risk in nursing students would be positively and significantly associated with academic burnout syndrome and with symptoms of depression, anxiety, and stress; while it would be negatively related to academic engagement. It was also postulated that a multiple regression model with these three predictors would significantly explain the variability of suicide risk in this university population.

## Methods

### Ethics statement

The protocol was approved by the Ethics and Scientific Research Committee of the sponsoring institution (Folio: 239/2019), in accordance with the Declaration of Helsinki. Data collection was conducted between April and August 2023 using classroom-administered surveys via Google Forms, with authorization from the classroom teachers. All participants signed an electronic informed consent form. Participation was voluntary, anonymous, and without monetary incentives. A STROBE Statement, which is a checklist of items that should be included in reports of observational studies, is included in S1 Checklist.

### Participants

The target population consisted of 253 nursing students from a Chilean public university, enrolled in their first to fifth year of study. From this universe, a minimum sample size of 153 participants was estimated, with a 95% confidence level and a 5% margin of error. All 253 eligible nursing students were invited to participate. To increase statistical power, 171 students were ultimately surveyed through convenience sampling, of whom three cases were considered outliers for the variable of interest, suicide risk, and therefore excluded from the final analysis. The present study data comprised 168 participants.

### Instruments

All questionnaires were administered in Spanish. Chilean-validated Spanish versions were used for the Beck Hopelessness Scale (BHS), the Maslach Burnout Inventory–Student Survey (MBI-SS), and the Depression, Anxiety, and Stress Scale (DASS-21). The Spanish-language version of the Utrecht Work Engagement Scale (UWES), previously used in Spanish-speaking and Chilean university populations, was also administered [36–40]. No translation of the instruments was performed specifically for the present study.

1) Bio-sociodemographic questionnaire: This questionnaire collected demographic and academic information, including age, sex, marital status, parenthood, year of study, and employment status. It also assessed social and contextual variables, including financial difficulties, family problems, and perceived availability of support networks. Health-related behaviors included physical activity, recreational activities, sleep duration, and the frequency of alcohol, tobacco, marijuana, and other substance use. Finally, participants reported underlying health conditions and previous mental health diagnoses. These variables were analyzed individually and were not combined into a composite “healthy habits” score.
2) Beck Hopelessness Scale (BHS), Chilean Spanish version [36]: a 20-item dichotomous self-report measure of hopelessness. Hopelessness is considered an established psychological factor associated with suicidal thoughts and behaviors. In the present study, BHS scores were used as a screening indicator of elevated suicide-related risk rather than as a diagnostic measure of suicidality. Scores were classified as follows: normal/asymptomatic, 0–3; mild hopelessness, 4–8; moderate hopelessness, 9–14; and severe hopelessness, 15–20. Following the Chilean validation study, scores ≥ 9 were considered indicative of elevated suicide-related risk [36]. Consistent with established standards for psychological testing, this screening use should not be interpreted as equivalent to an individual clinical diagnosis or comprehensive assessment of suicidality [41]. The BHS does not directly assess suicidal ideation, suicide attempts, self-harm, or future suicidal behavior. Total reliability in this sample was good, *α* = 0.86; *ω* = 0.86.
3) Utrecht Work Engagement Scale (UWES), 17 items distributed across vigor (*α* = 0.78; *ω* = 0.78), dedication (*α* = 0.80; *ω* = 0.81), and absorption (*α* = 0.82; *ω* = 0.82). Responses were on a 7-point Likert scale (0 = never; 6 = every day) [37,38,42]. For descriptive analyses, item scores were averaged within each dimension and for total engagement, yielding mean scores ranging from 0 to 6; higher scores indicate greater academic engagement. Total reliability in the sample was good: *α* = 0.91; *ω* = 0.91.
4) Maslach Burnout Inventory Students Survey (MBI-SS), Chilean Spanish version [39]; measures academic burnout syndrome in three dimensions: emotional exhaustion (*α* = 0.87; *ω* = 0.87), cynicism (*α* = 0.64; *ω* = 0.65) and academic efficacy (*α* = 0.74; *ω* = 0.74). For descriptive analyses, scores were expressed as mean item scores for each dimension. Higher scores in emotional exhaustion and cynicism, together with lower scores in academic efficacy, indicate greater academic burnout. Total reliability in the sample was good: *α* = 0.82; *ω* = 0.81. A higher score in the first two or a lower score in the last reflects greater severity of the syndrome [39].
5) Depression, Anxiety, and Stress Scale-21 (DASS-21), Chilean Spanish version validated in university students [40]: a 21-item self-report instrument comprising three seven-item subscales assessing symptoms of depression, anxiety, and stress. The DASS-21 was selected because it provides a brief, simultaneous assessment of these three dimensions of psychological distress and has been validated among Chilean university students. In the present study, it was used to characterize symptom severity at the group level, not to establish psychiatric diagnoses or to directly assess suicidality. Depression showed α = 0.91 and ω = 0.91; anxiety, α = 0.87 and ω = 0.89; and stress, α = 0.85 and ω = 0.85. Established cut-off scores were used to classify symptom severity as normal, mild, moderate, severe, or extremely severe [40].

### Data analysis

A cross-sectional and correlational study was conducted. Statistical analyses were conducted using R, version 4.5.3 [43], and RStudio, version 2026.08.2 [44], yielding percentage distributions and measures of central tendency. Data normality was verified through visual inspection of normality plots and outlier detection using the IQR x 1.96 rule. From this sample, three cases were removed from the final analyses, as they scored extremely high (severe) on suicide risk, being beyond the IQR × 1.96 rule. These participants, who scored 13, 14, and 15 on the BHS scale, were consequently excluded from the final analyses.

Unreliable instrument indicators were excluded from the analyses (those with a corrected total item correlation below .30). For Cynicism, item 15, which loaded on the dimension, was found to be insufficiently reliable and was excluded from the analyses. For Emotional Exhaustion, item 14 was removed. Finaly, for the BHS, items 5,6,13,17,19, and 20 were excluded. Associations between variables were explored using Pearson’s correlation coefficient.

Multiple linear regression was applied to the main hypothesis. In this analysis, we used the total suicide risk score as the criterion variable. The hypothesized multiple linear regression analysis model included the following predictors: 1) academic engagement, 2) academic burnout syndrome, and 3) symptoms of depression, anxiety, and stress.

The initial regression model included all predictors specified a priori on theoretical grounds. Because several predictors did not contribute significantly to the model, a reduced model was subsequently examined as an exploratory analysis. Predictor removal was therefore not interpreted as confirmation or rejection of the original hypothesis, but as a secondary model-refinement procedure. The full hypothesized model and the reduced model were both considered when interpreting the findings.

## Results

### Descriptive statistics

The sample consisted of 168 nursing students, mostly women (80.95%), single (98.21%), and without children (98.81%). First-year (31.55%) and fourth-year (27.98%) students predominated, with ages mainly between 18 and 22 years (87.50%). Nearly a quarter of the participants were working (23.80%), while a similar proportion (23.21%) reported experiencing financial difficulties. 32.74% reported family problems and 11.90% reported lacking support networks. In terms of lifestyle habits, 54.17% did not engage in physical activity, 28.57% did not engage in recreational activities, and 39.89% slept less than or up to five hours a day (Table 1). Regarding preexisting conditions, 32.14% reported medical or psychological underlying conditions, and in more than 80.00% of these cases, symptoms of depression, anxiety, or stress were reported (See S2 Table for full descriptive statistics).

**Table 1:** Description of selected biosociodemographic variables of nursing students (N = 168).

*Variable*	*n (%)*	*Suicide risk (%)*
Age 17–19	59 (35.10)	16.95
Age 20–22	88 (52.40)	7.95
Age 23–25	19 (11.30)	5.26
26 or older	2 (1.20)	50.00
Female	136 (81.00)	13.24
Male	31 (18.50)	3.23
Non-binary	1 (0.60)	0.00
1st year	53 (31.55)	20.75
2nd year	33 (19.64)	9.09
3rd year	8 (4.76)	14.29
4th year	47 (28.00)	4.26
5th year	28 (16.70)	7.14
Family problems (Yes)	55 (32.70)	21.82
Family problems (No)	114 (67.50)	6.19
Economic Problems (Yes)	39 (23.20)	23.08
Economic Problems (No)	130 (77.40)	8.53
No social support	20 (11.90)	25.00
Informed social support	149 (88.70)	10.14
Pathology Yes)	54 (32.10)	16.67
Pathology (No)	115 (68.50)	39.47

Supplementary S2 Table provides detailed cross-tabulations of DASS-21, engagement and burnout dimensions.

### BHS-based elevated suicide-related risk

Using the predefined BHS cut-off of ≥ 9 points, 19 of the 168 participants (11.31%) were classified as presenting elevated suicide-related risk based on moderate-to-severe hopelessness. Among women, 13.2% met the predefined BHS threshold, compared with 3.23% of men. No non-binary participant met the predefined BHS threshold. Similarly, a higher proportion of participants meeting the predefined BHS threshold was observed among those who worked (15.00%) in comparison to those who did not (10.9%), as well as among participants who reported experiencing family problems (21.80%) in comparison to those who did not experience such problems (7.0%). The proportion meeting the predefined BHS threshold was also higher among participants who reported lacking support networks (25.00%) than among those who did have such support (10.14%), as presented in Table 1. The participants who slept between three and five hours (38.10%) had a higher risk of suicide than those who slept between six and eight hours (58.33%). Similarly, the participants with underlying medical conditions (32.14%) had nearly double the suicide risk compared to participants with no declared underlying medical conditions. In addition, all participants who declared experiencing either depressive or anxiety symptoms qualified for suicide risk.

### Academic engagement and burnout

Academic engagement scores were expressed as mean item scores on a 0–6 scale. Dedication showed the highest mean score (*M* = 4.50, *SD* = 1.01), followed by absorption (*M* = 3.53, *SD* = 1.19) and vigor (*M* = 3.21, *SD* = 1.18). The mean total engagement score was 3.79 (*SD* = 1.01). According to the percentile-based interpretative framework proposed by Schaufeli and Bakker, the observed mean scores were within the average range [42]; scores within ±1 SD of the reference mean can be considered within the average range (Table 2).Engagement levels were higher for first-year students, those who worked, had no family problems, engaged in physical activity, or consumed alcohol and marijuana occasionally. This bivariate pattern indicates an inverse association between academic engagement and BHS scores, consistent with the subsequent correlation analysis.

**Table 2:** Descriptive statistics of academic engagement and academic burnout syndrome in nursing students (N = 168).

		*Mean*	*Moda*	*SD*	*Minimum*	*Maximum*	*Percentiles*		
							*p25*	*p50*	*p75*
Academic engagement	Vigor	3.21	3.40	1.18	0.40	5.80	2.20	3.40	4.00
	Dedication	4.50	4.55	1.01	0.33	6.00	4.17	4.67	5.17
	Absorption	3.53	4.17	1.19	0.33	6.00	2.67	3.67	4.33
	Total Engagement	3.79	4.00	1.01	1.24	5.94	3.18	3.98	4.59
Academic Burnout Syndrome	Cynicism	1.52	1.00	1.00	0.00	4.33	0.67	1.33	2.33
	Emotional Exhaustion	3.98	5.00	1.23	1.00	6.00	3.00	4.08	5.00
	Academic Efficacy	2.04	1.88	0.90	0.13	5.13	1.38	2.00	2.63
	Total ABS	2.56	2.63	0.79	0.58	4.27	2.00	2.58	3.11

Regarding Academic Burnout, the sample presented average levels of Emotional Exhaustion and Cynicism, and markedly lower scores of academic efficacy compared to similar populations of students of nursing and health workers [6,45]

### Depression, anxiety, and stress (DASS-21)

Although more than 75% of the students presented either depressive, anxiety, or stress symptoms, it was noticeable that 3.6% of the nursing students presented severe to extremely severe depressive symptoms, and that 30.2% presented severe to extremely severe anxiety symptoms. Moreover, neither severe nor extremely severe values for Stress were present in the study sample. Overall, women, single students, and first-year students presented more symptomatology. Moreover, comparable levels were observed in students who worked, experienced family or financial problems, lacked support networks, or slept less than five hours. There were strong and positive correlations between marihuana consumption and the consumption of other substances in the sample, all of which were statistically significant: Alcohol, *r*_*s*_ (168) = .40, *p* < .001; Tobacco, *r*_*s*_ (168) = .31, *p* < .001; and LSD, *r*_*s*_ (168) = .24, *p* < .01.

### Inferential statistics

Suicide risk correlated negatively with vigor, dedication, and absorption in academic engagement, and positively with academic burnout syndrome dimensions and with depression, anxiety, and stress (Fig 1). The strongest association was with depression (*r* = 0.64; *p* < 0.001, see Fig 1).

**Fig 1:**
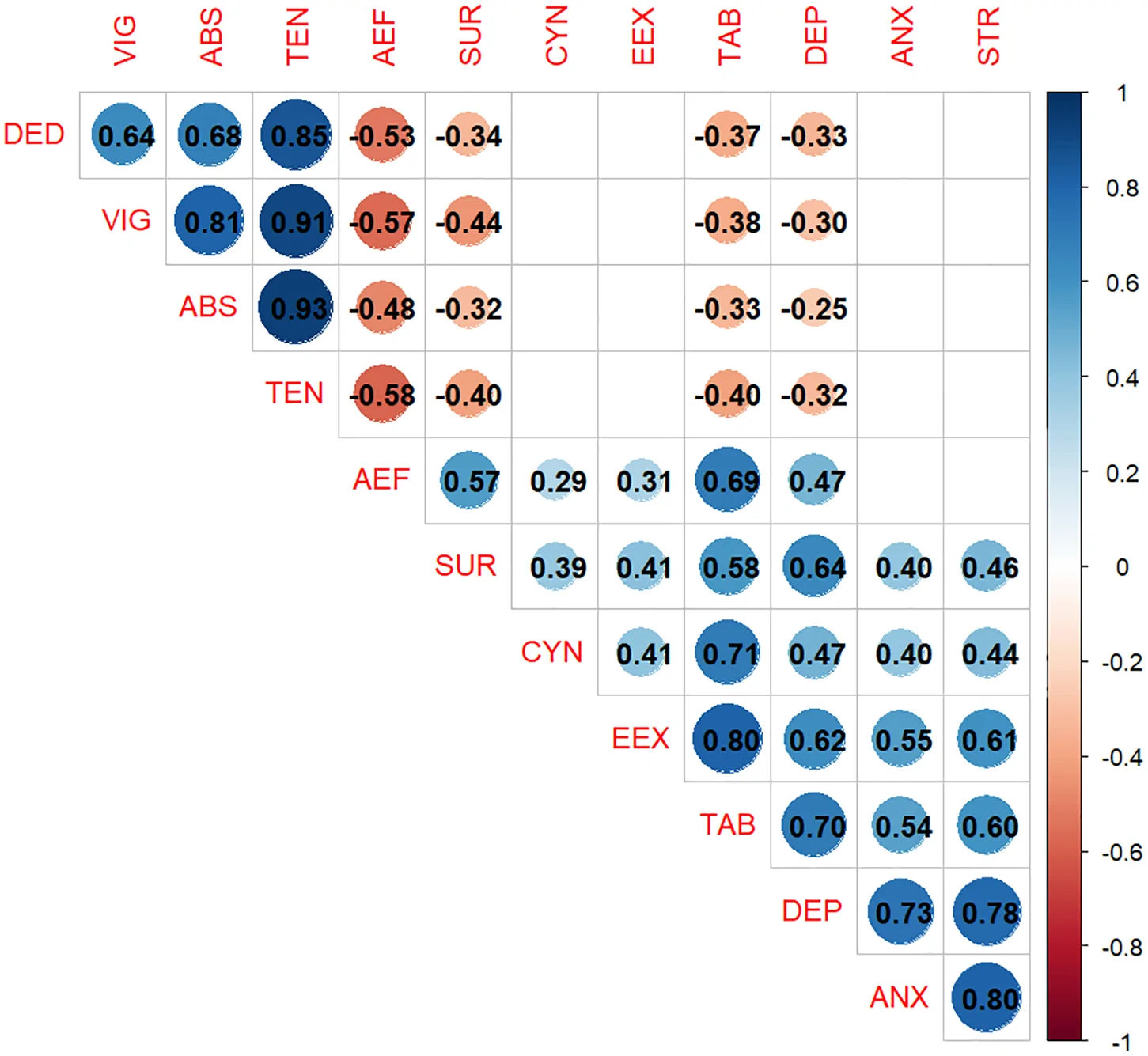
Correlation matrix for suicide risk in nursing students. DED = Dedication; VIG = Vigor; ABS = Absorption; TEN = Total Engagement; AEF = Academic Efficacy; SUR = Suicide Risk; CYN = Cynicism; EEX = Emotional Exhaustion; TAB = Total Academic Burnout; DEP = Depression; ANX = Anxiety; STR = Stress.

In assessing parametric requirements for linear regression among nursing students, no Cook’s distance values exceeded 0.24, suggesting they would not bias the model. To ensure the validity of the model, the independence of the residuals was verified using the Durbin-Watson statistic, yielding a value of 2.25, indicating the absence of autocorrelation. Potential collinearity among the independent variables was assessed using the Variance Inflation Factor (VIF) and Tolerance values.

For the predictor ‘Depression’, a VIF of 1.29 and a Tolerance of 0.78 were obtained. For the predictor ‘Vigor’, a VIF of 1.49 was obtained, Tolerance: 0.67. For the predictor ‘Academic Efficacy’, a VIF of 1.78 was obtained, Tolerance: 0.56. Finally, for the interaction between Depression and Vigor, a VIF of 1.02 and Tolerance: 0.98 were obtained. These values are clearly far from the thresholds indicating problematic collinearity for VIF and Tolerance, respectively (VIF > 5; Tolerance < .20), which were absent among the predictors tested in the multiple regression model. Homoscedasticity was confirmed through graphical analysis of the residuals and specific statistical tests, and linearity was verified by evaluating the relationships between the independent variables and the dependent variable, ensuring they were linear.

The initially hypothesized model included academic engagement, academic burnout, and DASS-21 dimensions. A secondary reduced model was subsequently examined to identify the predictors contributing most strongly to BHS scores. This reduced model should be interpreted as exploratory rather than a priori confirmatory. The reduced model comprised Depression, Vigor, Academic Efficacy, and the Depression × Vigor interaction. The results of this analysis revealed that the regression model with the predictors explained 54% of the variability in suicide risk (*Adj. r*² = 0.54). All coefficients are presented in Table 3, which indicates that Depression, Vigor, Academic Efficacy, and the interaction between Depression and Vigor are significant for understanding and predicting suicide risk in the studied population with a very large effect size (*f²* = 1.10). Cohen’s *f* partial coefficient were: 1) Depression (0.81), which is interpreted as large effect size, 2) Vigor (0.25), which is interpreted as medium effect size, 3) Academic Efficacy (0.22), which is interpreted as a low to medium effect size, and 4) the interaction between Depression and Vigor (0.22), which is interpreted as a low to medium effect size as well.

**Table 3:** Multiple linear regression model for factors associated with suicide risk in nursing students (N = 168).

*Predictors*	*F*	*df1*	*df2*	*R*²	*Adj.R*²	*B*	*Std. Error*	*t*	*p*	*Cohen’s f (partials)*	*Cohen’s f²*
	*Constant*					−3.37**	1.23	−2.74	< .001		
Depression	48.69	4.00	162.00	0.55	0.54	4.04***	0.61	6.61		0.81	1.10
Vigor						0.63*	0.30	2.12		0.25	
Academic Efficacy						0.88**	0.26	3.44		0.22	
Depression*Vigor						−0.69***	0.17	−4.00		0.22	

*N* = 168. *F* = Fisher Statistic; *df1* = degrees of freedom of the predictors; *df2* = degrees of freedom of the sample; *R²* = Coefficient of determination; *Adj. R²*= Adjusted R². The dependent variable corresponds to suicide risk (SR); *Std. Error* = Standard Error. * *p* < .05, ** *p* < .01, *** *p* < .001.

## Discussion

This study sought to relate risk and protective factors for mental health in nursing students in the post-pandemic context. It significantly related the predictor variables academic engagement, academic burnout syndrome, and depression, anxiety, and stress to the criterion variable suicide risk. Weak and moderate negative associations were found between suicide risk and the academic engagement subscales of vigor, dedication, and absorption. Likewise, strong and positive associations were observed between suicide risk, the academic efficacy dimension, and the depression dimensions. In addition, moderate positive associations were reported between the Cynicism and emotional exhaustion subscales from academic burnout syndrome, as well as anxiety and stress. This confirmed the study’s first hypothesis and is consistent with prior research [6,27,32].

### Proportion of students meeting the BHS threshold for elevated suicide-related risk

During the post-pandemic academic normalization period, 11.31% of participants met the predefined BHS threshold for elevated suicide-related risk, corresponding to moderate-to-severe hopelessness, which was comparable to what was reported in a recent review that included healthcare students (14.09%) [46]. This proportion represents a decrease compared with previous studies conducted during the phase immediately following lockdown [8,33]. However, this proportion is still higher than figures documented during lockdown in Latin American countries [6]. However, national statistics for Chilean young people aged 18–24 refer to suicide mortality rates rather than screening-based prevalence estimates and are therefore not directly comparable with the present BHS-based proportion [47], especially when considering the moderate sample size of the present study. Overall, the findings are consistent with previous evidence suggesting that the nursing student population is more susceptible to suicide risk [1,8,32,48].

Nursing students may be exposed during clinical training to emotionally demanding patient-care experiences, including caring for individuals experiencing pain, severe illness, suffering, and death [13,22]. Such exposure may contribute to psychological strain alongside the academic demands of nursing education. However, students in the early years of training are generally less exposed to these harsh, clinically demanding settings than students in their final years of study. Nevertheless, the proportion of students meeting the predefined BHS threshold for suicide risk was higher in the initial years of nursing training, raising a relevant question: How will their stress levels be affected once they begin their clinical practice? This finding aligns with studies reporting lower levels of suicide-related risk as age increases in this population [8,49], and it is consistent with past research in the general population [49].

It is a well-known fact that women attempt suicide up to three times more than men, although men have higher rates of completed suicide [15]. This is worth considering in light of the present study, as approximately 81% of the sample in the study comprised women, 16% of whom qualified for elevated suicide-related risk by the BHS threshold. This proportion was similar to the 10.0% reported in a previous local study conducted during the COVID-19 pandemic [50], but lower than the 19.3% reported during the return to face-to-face academic activities [8]. These differences could reflect progressive adaptation to the post-pandemic context and the influence of protective factors, such as academic engagement.

### Academic engagement and burnout

Regarding academic engagement, the sample showed an overall mean score of 3.78 (*SD* = 1.03) on the 0–6 UWES scale, with dedication presenting the highest mean (*M* = 4.55, *SD* = 1.01), followed by absorption (*M* = 3.52, *SD* = 1.22) and vigor (*M* = 3.19, *SD* = 1.21). According to the interpretative framework proposed by Schaufeli and Bakker (2004), these scores are broadly consistent with an average level of engagement [42]. These findings differ from those reported in previous studies conducted with other university populations during the pandemic [35,51], suggesting that academic engagement may be influenced by the educational and environmental setting in which students are confined.

Engagement scores also varied across the study of career progression, with higher values observed among students in the earlier years of training. This pattern is partially consistent with previous evidence suggesting that academic engagement may be particularly pronounced at specific stages of university training, i.e., at the beginning and at the end of the study of career [51]. However, given the cross-sectional design, these differences should be interpreted with caution.

Regarding academic burnout, emotional exhaustion showed the highest mean score (*M* = 3.98, *SD* = 1.23), followed by academic efficacy (*M* = 2.04, *SD* = 0.90) and cynicism (*M* = 1.52, *SD* = 1.00). In the MBI-SS, higher emotional exhaustion and cynicism scores and lower academic efficacy scores indicate greater burnout severity. The comparatively low academic efficacy observed in this sample is particularly relevant and is consistent with previous evidence showing substantial academic burnout among nursing students during and after the pandemic [6,14].

### Depression, anxiety, and stress

Likewise, markedly high levels of symptoms of depression, anxiety, and stress were identified in first-year students. When comparing these findings with research conducted in a pre-pandemic context in a similar academic population, whose rates fluctuated between 49% and 64% [51], a substantial increase is evident. In this study, the first-year prevalences were 84.91% for depression, 90.57% for anxiety, and 79.25% for stress, figures far higher than those reported by an umbrella review, which reported prevalences of 29%, 29%, and 27% in nursing students [14]. These rates are like those observed during the pandemic in Mexican students (85.8% depression; 84% anxiety; 77.4% stress) [21]. In addition, the highest prevalences were concentrated in the first and third years and in the 18–22 age range, coinciding with Chilean studies that identify this age group as the most vulnerable [52]. This suggests a possible impact of the current context on mental health in nursing students. In addition, high levels of depression, anxiety, and stress were observed before, during, and after the pandemic.

Employed participants reported comparable levels of Depression, Anxiety and Stress to those who were unemployed, suggesting that being employed does not have an impact on these symptoms. In the family sphere, family problems were highly associated with higher levels of depression (83.64%), anxiety (92.73%), and stress (89.09%).

Regarding physical and recreational activities, which are identified as protective factors for mental health [53], our research found no significant differences in DASS levels across these activities, with DASS levels remaining high regardless of whether these activities are performed. However, it can be observed that respondents who do not engage in physical activities have more depressive symptoms (79.12%), which is consistent with the literature that points to depression as an indicator closely linked to suicide risk [18].

In terms of health-related behaviors, it was observed that students consumed alcohol (13.69%), tobacco (14.88%), and marijuana (7.74%) at least once a week. Those who drank alcohol weekly showed high prevalences of anxiety (82.61%) and stress (73.91%), which is consistent with a study conducted with health students in Brazil, where a significant correlation was reported between alcohol consumption and symptoms of stress and anxiety [54]. Similarly, students who used marijuana weekly had a prevalence of 100% for anxiety and 92.31% for stress. Although the present data are cross-sectional, longitudinal evidence indicates that cannabis use during adolescence is associated with a higher subsequent risk of depression and suicidal behavior in young adulthood [55]. This underscores the need to raise awareness among the university community about the risks of harmful substance use and its long-term consequences for MH.

It is important to note that students without a mental health diagnosis still presented high levels of Depressive, Anxiety and Stress symptoms from the DASS scale (65.04%; 79.67%; 68.29%, respectively), and that there was a significant difference in incidence compared to those who disclosed or were aware of their diagnosis. This suggests that students are unaware of the severity of their symptoms and therefore do not recognize the need to seek mental health counseling or psychotherapy. This lack of recognition increases the risk of these symptoms worsening if early diagnosis and treatment are unavailable. Furthermore, moderate and strong positive correlations were observed between Depressive, Anxiety, Stress from DASS, and Suicide Risk. It is therefore crucial to identify students’ mental health status at an early stage, to promote psychological wellbeing, preventing further risks, and fostering a psychologically supportive environment, if required.

The second hypothesis stated that the predictor variables in the proposed multiple regression model (i.e., academic engagement, academic burnout syndrome, depression, anxiety, and stress) would significantly associate with suicide risk. “The findings provided support for this hypothesis, as Depression, Vigor, Academic Efficacy, and the interaction between Depression and Vigor were significantly associated with suicide risk. The model accounted for 54% of the variance in suicide risk (*R²* = 0.55; adjusted *R²* = 0.54), representing a large effect size. The direction of the effects for Depression, Vigor, and Academic Efficacy on Suicide Risk was positive, meaning that as participants’ scores on these variables were higher, their suicide risk increased. The magnitude of these effects was large for Depression and Vigor, and medium to large for Academic Efficacy and for the interaction between Depression and Vigor. This means that Depressive symptoms (i.e., Depression), high levels of energy, persistence, and the willingness to engage in academic tasks (i.e., Vigor), and the student’s self-perception of competence, success, and effectiveness in their academic labor (i.e., Academic Efficacy) were positively associated with BHS scores as main effects in the reduced regression model. Moreover, the direction of the interaction on suicide risk was negative, meaning the combined effects of these predictors reduced suicide risk, either by the attenuation of depressive symptomatology through high levels of energy, persistence, and the willingness to engage in academic tasks, or through the reduction of potentially iatrogenic effects from high excessive vigor on the psychological wellbeing of the students examined through depressive symptoms. These results align closely with the literature, as depression was confirmed as a robust associated factor of suicide risk, in line with evidence from a large study of university students during the COVID-19 pandemic [56]. Similarly, the vigor dimension of academic engagement [34] was found to be a protective factor before and after the pandemic, which was partially corroborated by the interaction term, although the standalone main effect of Vigor was found to increase suicidal risk.

This study is not exempt from limitations. First, given the cross-sectional nature of the study design, the results from the multiple regression model should be interpreted with caution and treated as hypothesis-generating rather than causal. Second, the sample comprised mainly women, which prevented comparisons across genders and limited the generalizability of the findings. It is recommended that future research work with more balanced samples in terms of sex and gender. Third, previous suicidal ideation, suicide attempts, and self-harm behaviors were not assessed, which prevented the potential assessment of future suicidality. Accordingly, the BHS-based indicator used in this study should be interpreted as a screening measure based on hopelessness and not as a comprehensive assessment of suicidality, which should include pathognomonic symptoms and signs, such as suicidal thoughts and related behaviors, in addition to hopelessness. Fourth, the limited evidence available in Chile on the interaction between academic engagement, academic burnout syndrome, depression, anxiety, stress, and suicide risk makes international comparison and understanding of the sociocultural particularities of this population difficult. Expanding research in local and multicenter contexts would help advance a more comprehensive and culturally relevant model.

## Conclusion

This study confirmed that suicide risk among nursing students is a rather complex phenomenon that is far from being explained only through traditional clinical factors, as it can be largely explained by the interaction between personal resources, academic demands, and the students’ psychological wellbeing. In the present study, depressive symptoms were the strongest predictor of suicide risk; followed by Vigor, a dimension depicting academic engagement; Academic Efficacy, a dimension protecting from academic burnout, which was found in the sample; and by the interaction between Depressive symptoms and Vigor, which was found to reduce suicide risk in the model. The findings revealed a paradox, as academic motivation can erode psychological well-being and increase suicide risk when a cognitive or emotional over-involvement in academic tasks is present. This result reinforces the need for a comprehensive approach to higher education that promotes protective resources, encourages healthy boundaries, and offers psychosocial support, so that academic commitment enhances personal and professional development. Future research should replicate the study’s findings using longitudinal designs and a more heterogeneous sample, which may allow for a better understanding of the dynamics among engagement, burnout, and suicide risk. In addition, this is expected to inform more effective institutional policies to promote student psychological wellbeing.

## Supporting information

S1 Checklist STROBE Statement—checklist of items that should be included in reports of observational studies. (DOCX)

S2 Table Bio-sociodemographic characteristics, BHS-based elevated suicide-related risk, DASS-21 symptom levels, academic engagement, and academic burnout among nursing students included in the final analysis (N = 168). (DOCX)
