# Supplementary material for: Suicide risk factors in nursing students: Depressive symptoms, academic burnout and academic efficacy after the pandemic
Source: PLOS Ment Health. 2026 Nov 10;3(11):e0000734. doi: 10.1371/journal.pmen.0000734 (PMC13750606; doi:10.1371/journal.pmen.0000734)
Supplement: Supplementary file 1 [file pmen.0000734.s001.docx]

**S1 Checklist**. STROBE Statement—checklist of items that should be included in reports of observational studies

|  | Item No. | Recommendation | Page  No. | Relevant text from manuscript |
| --- | --- | --- | --- | --- |
| **Title and abstract** | 1 | (*a*) Indicate the study’s design with a commonly used term in the title or the abstract | 1 | Methods: Cross-sectional and correlational study of 168 nursing students at a Chilean public university.” |
|  |  | (*b*) Provide in the abstract an informative and balanced summary of what was done and what was found | 1 | Objective: To analyze the prevalence and factors associated with suicide risk in nursing students in the post-pandemic context, considering academic burnout, academic engagement, and symptoms of depression, anxiety, and stress as predictors.  Methods: Cross-sectional and correlational study of 168 nursing students at a public university in Chile. Validated instruments were applied: Beck Hopelessness Scale, Utrecht Work Engagement Scale, Maslach Burnout Inventory–Student Survey, and DASS-21. Descriptive analyses, Pearson correlations, and multiple linear regression were performed. Statistical assumptions, collinearity, and testing for residual independence were assessed.  Results: A total of 11.3% of students met the predefined BHS threshold for elevated suicide-related risk. In the same sample, symptoms of depression, anxiety, and stress showed high prevalence rates. Suicide risk was negatively associated with vigor and dedication, while positively associated with low academic efficacy and depression. The regression model explained 54% of the variance in suicide risk (Adj.R² = 0.54; p < 0.001), with Depression (β = 4.04), Vigor (β = 0.63), Academic Efficacy (β = 0.88), and the interaction between Depression and Vigor (β = -0.69) being significant predictors in the model.  Conclusions: Suicide risk in nursing students transcends traditional clinical factors, as it is shaped by the interaction between personal resources and mental health symptoms. These findings underscore the need for prevention and psychosocial support programs in nursing schools. |
| Introduction | | | |  |
| Background/rationale | 2 | Explain the scientific background and rationale for the investigation being reported | 2-4 | Suicide risk is a significant public health issue, particularly among university students. Nursing students are more vulnerable to suicide risk due to the high academic and emotional demands from their training.  The COVID-19 pandemic reported a 25% increase in the global prevalence of anxiety and depression.  Academic engagement has been described as a protective factor against psychological maladjustment in university students. During the pandemic, it also acted as a buffer against stress-related symptoms and adverse mental health outcomes. This raises the question of its role in the post-pandemic period, as its interaction with academic burnout syndrome and depression, anxiety, and stress to explain suicide risk remains largely unexplored. |
| Objectives | 3 | State specific objectives, including any prespecified hypotheses | 4-5 | The purpose of this study was to identify factors associated with suicide risk in nursing students at a public university in the post-pandemic context.  The hypothesis was proposed that suicide risk in nursing students would be positively and significantly associated with academic burnout syndrome and with symptoms of depression, anxiety, and stress; while it would be negatively related to academic engagement. |
| Methods | | | |  |
| Study design | 4 | Present key elements of study design early in the paper | 5 | A cross-sectional and correlational study was conducted. |
| Setting | 5 | Describe the setting, locations, and relevant dates, including periods of recruitment, exposure, follow-up, and data collection | 5-6 | The target population consisted of 253 nursing students from a Chilean public university, enrolled in their first to fifth year of study.  Data collection was conducted between April and August 2023, using classroom-administered surveys via Google Forms. |
| Participants | 6 | (*a*) *Cohort study*—Give the eligibility criteria, and the sources and methods of selection of participants. Describe methods of follow-up  *Case-control study*—Give the eligibility criteria, and the sources and methods of case ascertainment and control selection. Give the rationale for the choice of cases and controls  *Cross-sectional study*—Give the eligibility criteria, and the sources and methods of selection of participants | 5 | The target population consisted of 253 nursing students at a Chilean public university, enrolled in their first to fifth years of study. Eligible participants were undergraduate nursing students enrolled in their first to fifth years who agreed to participate voluntarily and completed the study questionnaires. Students who declined to participate or who submitted incomplete questionnaires were excluded from the analysis. From this population, a minimum sample size of 153 participants was estimated, assuming a 95% confidence level and a 5% margin of error. To increase statistical power, 171 students were ultimately surveyed. From this sample, three cases were considered as outliers. Hence, a sample of 168 cases was used for data analysis. |
|  |  | (*b*) *Cohort study*—For matched studies, give matching criteria and number of exposed and unexposed  *Case-control study*—For matched studies, give matching criteria and the number of controls per case | --- | Not applicable |
| Variables | 7 | Clearly define all outcomes, exposures, predictors, potential confounders, and effect modifiers. Give diagnostic criteria, if applicable | 6-7 | In this analysis, we used the total suicide risk score as the criterion variable.  The multiple linear regression analysis model included the following predictors: 1) academic engagement, 2) academic burnout syndrome, and 3) symptoms of depression, anxiety, and stress. |
| Data sources/ measurement | 8* | For each variable of interest, give sources of data and details of methods of assessment (measurement). Describe comparability of assessment methods if there is more than one group | 6-7 | Beck Hopelessness Scale (BHS): 20 dichotomous items measuring suicide risk.  Utrecht Work Engagement Scale (UWES), 17 items distributed across vigor, dedication, and absorption.  Maslach Burnout Inventory Students Survey (MBI-SS) measures academic burnout syndrome.  The Depression, Anxiety, and Stress Scale number 21 (DASS-21) comprises three subscales of seven items each. |
| Bias | 9 | Describe any efforts to address potential sources of bias | 7 | To minimize reporting bias, participation was anonymous and voluntary, and all questionnaires were completed independently using previously validated Chilean versions. However, because all variables were assessed via self-report measures, some degree of information bias cannot be entirely ruled out. |
| Study size | 10 | Explain how the study size was arrived at | 5 | From this universe, a minimum sample size of 153 participants was estimated, with a 95% confidence level and a 5% margin of error. To increase statistical power, 171 students were ultimately surveyed. |

Continued on next page

| Quantitative variables | 11 | Explain how quantitative variables were handled in the analysis. If applicable, describe which groupings were chosen and why | 6-7 | A score ≥ 9 predicts suicidal behavior. (BHS). Its cut-off points classify symptoms as normal, mild, moderate, severe or extremely severe (DASS-21). In this analysis, we used the total suicide risk score as the criterion variable. |
| --- | --- | --- | --- | --- |
| Statistical methods | 12 | (*a*) Describe all statistical methods, including those used to control for confounding | 7 | Statistical analysis was performed in R (version 4.5.3) and RStudio (version 2026.08.2), yielding percentage distributions and measures of central tendency. Data normality was verified using normality tests and visual inspection. Associations between variables were explored using Pearson's correlation coefficient. Multiple linear regression was applied to the main hypothesis. |
|  |  | (*b*) Describe any methods used to examine subgroups and interactions | ---- | Not applicable |
|  |  | (*c*) Explain how missing data were addressed |  | No missing data were detected in the final dataset; therefore, it was not necessary to apply imputation procedures or exclude cases for the statistical analyses. |
|  |  | (*d*) *Cohort study*—If applicable, explain how loss to follow-up was addressed  *Case-control study*—If applicable, explain how matching of cases and controls was addressed  *Cross-sectional study*—If applicable, describe analytical methods taking account of sampling strategy | ---- | Not applicable |
|  |  | (*e*) Describe any sensitivity analyses | --- | No sensitivity analyses were conducted. |
| Results | | | | |
| Participants | 13* | (a) Report numbers of individuals at each stage of study—e.g. numbers potentially eligible, examined for eligibility, confirmed eligible, included in the study, completing follow-up, and analyzed | 8 | Of the 253 eligible students, 171 agreed to participate and completed the questionnaires. 168 were included in final data analysis. |
|  |  | (b) Give reasons for non-participation at each stage | 5, 8 | Students who declined to participate or who submitted incomplete questionnaires were excluded from the analysis. |
|  |  | (c) Consider use of a flow diagram | --- | Not applicable |
| Descriptive data | 14* | (a) Give characteristics of study participants (e.g. demographic, clinical, social) and information on exposures and potential confounders | 8-9 | The sample consisted predominantly of women (81.00%), single students (98.21%), and students without children (98.81%). |
|  |  | (b) Indicate number of participants with missing data for each variable of interest | 8 | All variables included in the analysis were complete, and no missing observations were identified. |
|  |  | (c) *Cohort study*—Summarize follow-up time (e.g., average and total amount) | --- | Not applicable |
| Outcome data | 15* | *Cohort study*—Report numbers of outcome events or summary measures over time | 8–9 | The total suicide risk (moderate + severe) was 11.3%. |
|  |  | *Case-control study—*Report numbers in each exposure category, or summary measures of exposure |  |  |
|  |  | *Cross-sectional study—*Report numbers of outcome events or summary measures |  |  |
| Main results | 16 | (*a*) Give unadjusted estimates and, if applicable, confounder-adjusted estimates and their precision (e.g., 95% confidence interval). Make clear which confounders were adjusted for and why they were included | 11–12 | The final multiple linear regression model was statistically significant (*F* (4,162) = 48.69, p < 0.001) and explained 55% of the variance in suicide risk (adjusted *R²* = 0.54). Depression, vigor, and academic efficacy were positively associated with suicide risk, whereas the combined effect of vigor and depression decreased suicide risk. |
|  |  | (*b*) Report category boundaries when continuous variables were categorized | 6 | A score ≥ 9 predicts suicidal behavior for BHS. |
|  |  | (*c*) If relevant, consider translating estimates of relative risk into absolute risk for a meaningful time period | --- | Not applicable |

Continued on next page

| Other analyses | 17 | Report other analyses done—e.g., analyses of subgroups and interactions, and sensitivity analyses | 11 | To ensure the model's validity, the independence of the residuals was verified using the Durbin-Watson statistics. Potential collinearity was assessed using the Variance Inflation Factor (VIF) and Tolerance Values. |
| --- | --- | --- | --- | --- |
| Discussion | | | | |
| Key results | 18 | Summarize key results with reference to study objectives | 12-13 | This study sought to relate risk and protective factors for mental health in nursing students in the post-pandemic context. The study ought to analyze the prevalence and factors associated with suicide risk in nursing students in the post-pandemic context, considering academic burnout, academic engagement, and symptoms of depression, anxiety, and stress as predictors.  A total of 11.3% of students met the predefined BHS threshold for elevated suicide-related risk. In the same sample, symptoms of depression, anxiety, and stress showed high prevalence rates. Suicide risk was negatively associated with vigor and dedication, while positively associated with low academic efficacy and depression. The regression model explained 54% of the variance in suicide risk (Adj.R² = 0.54; p < 0.001), with Depression (β = 4.04), Vigor (β = 0.63), Academic Efficacy (β = 0.88), and the interaction between Depression and Vigor (β = -0.69) being significant predictors in the model. Suicide risk in nursing students transcends traditional clinical factors, as it is shaped by the interaction between personal resources and mental health symptoms. These findings underscore the need for prevention and psychosocial support programs in nursing schools. |
| Limitations | 19 | Discuss limitations of the study, considering sources of potential bias or imprecision. Discuss both direction and magnitude of any potential bias | 17 | This study is not exempt from limitations. First, given the cross-sectional nature of the study design, the results from the multiple regression model should be interpreted with caution and treated as hypothesis-generating rather than causal. Second, the sample comprised mainly women, which prevented comparisons across genders and limited the generalizability of the findings. It is recommended that future research work with more balanced samples in terms of sex and gender. Third, previous suicidal ideation, suicide attempts, and self-harm behaviors were not assessed, which prevented the potential assessment of future suicidality. Accordingly, the BHS-based indicator used in this study should be interpreted as a screening measure based on hopelessness and not as a comprehensive assessment of suicidality, which should include pathognomonic symptoms and signs, such as suicidal thoughts and related behaviors, in addition to hopelessness. Fourth, the limited evidence available in Chile on the interaction between academic engagement, academic burnout syndrome, depression, anxiety, stress, and suicide risk makes international comparison and understanding of the sociocultural particularities of this population difficult. Expanding research in local and multicenter contexts would help advance a more comprehensive and culturally relevant model. |
| Interpretation | 20 | Give a cautious overall interpretation of results considering objectives, limitations, multiplicity of analyses, results from similar studies, and other relevant evidence | 13-18 | The results must be interpreted as exploratory given the cross-sectional nature of the data and the relatively small sample size, in concordance with the limitations presented in the previous section. |
| Generalizability | 21 | Discuss the generalizability (external validity) of the study results | 17 | The sample was predominantly women, which prevented comparisons across genders and limited the generalizability of the findings. |
| Other information | |  | | |
| Funding | 22 | Give the source of funding and the role of the funders for the present study and, if applicable, for the original study on which the present article is based | 19 | The study did not receive any funding for the design, execution, collection, or analysis of the research presented in the current manuscript. |

*Give information separately for cases and controls in case-control studies and, if applicable, for exposed and unexposed groups in cohort and cross-sectional studies.

**Note:** An Explanation and Elaboration article discusses each checklist item and gives methodological background and published examples of transparent reporting. The STROBE checklist is best used in conjunction with this article (freely available on the websites of PLoS Medicine at http://www.plosmedicine.org/, Annals of Internal Medicine at http://www.annals.org/, and Epidemiology at http://www.epidem.com/). Information on the STROBE Initiative is available at [www.strobe-statement.org](http://www.strobe-statement.org).

Reference

von Elm E, Altman DG, Egger M, Pocock SJ, Gøtzsche PC, Vandenbroucke JP; STROBE Initiative. **The Strengthening the Reporting of Observational Studies in Epidemiology (STROBE)statement: guidelines for reporting observational studies.** [J Clin Epidemiol. 2008 Apr](http://download.journals.elsevierhealth.com/pdfs/journals/0895-4356/PIIS0895435607004362.pdf);61(4):344-9. PMID: 18313558
