# Supplementary material for: Suicide risk factors in nursing students: Depressive symptoms, academic burnout and academic efficacy after the pandemic
Source: PLOS Ment Health. 2026 Nov 10;3(11):e0000734. doi: 10.1371/journal.pmen.0000734 (PMC13750606; doi:10.1371/journal.pmen.0000734)
Supplement: Supplementary file 2 [file pmen.0000734.s002.docx]

S1 Table. Bio-sociodemographic characteristics, BHS-based elevated suicide-related risk, DASS-21 symptom levels, academic engagement, and academic burnout among nursing students included in the final analysis (N = 168).

| Bio-sociodemographic variables | | | Sample | | BHS-based elevated suicide-related risk | | Depression. Anxiety. Stress | | | | | | Academic engagement | | | | | | | | Academic burnout syndrome | | | | | |
| --- | --- | --- | --- | --- | --- | --- | --- | --- | --- | --- | --- | --- | --- | --- | --- | --- | --- | --- | --- | --- | --- | --- | --- | --- | --- | --- |
|  |  |  |  |  |  |  | Symptomatic levels | | | | | | High / elevated levels | | | | | | | | Sub-scales | | | | | |
|  |  |  | *n* | % | n | % | Depression n | % | Anxiety n | % | Stress n | % | Vigour n | % | Dedication n | % | Absorption n | % | Total n | % | Cynicism high n | % | Emotional exhaustion high n | % | Personal nulnilment low n | % |
| Demographics | Age ranges | 18–19 | 59 | 35.12 | 10 | 16.95 | 48 | 81.36 | 52 | 88.14 | 47 | 79.66 | 25 | 42.37 | 49 | 83.05 | 30 | 50.85 | 35 | 59.32 | 40 | 67.80 | 41 | 69.49 | 56 | 94.92 |
|  |  | 20–22 | 88 | 52.38 | 7 | 7.95 | 57 | 64.77 | 74 | 84.09 | 67 | 76.14 | 42 | 47.73 | 82 | 93.18 | 52 | 59.09 | 57 | 64.77 | 39 | 44.32 | 62 | 70.45 | 88 | 100.00 |
|  |  | 23–25 | 19 | 11.31 | 1 | 5.26 | 11 | 57.89 | 14 | 73.68 | 11 | 57.89 | 6 | 31.58 | 17 | 89.47 | 8 | 42.11 | 11 | 57.89 | 7 | 36.84 | 9 | 47.37 | 19 | 100.00 |
|  |  | 26 and older | 2 | 1.19 | 1 | 50.00 | 2 | 100.00 | 1 | 50.00 | 1 | 50.00 | 0 | 0.00 | 1 | 50.00 | 0 | 0.00 | 0 | 0.00 | 2 | 100.00 | 2 | 100.00 | 1 | 50.00 |
|  | Gender | female | 136 | 80.95 | 18 | 13.24 | 97 | 71.32 | 114 | 83.82 | 107 | 78.68 | 64 | 47.06 | 121 | 88.97 | 80 | 58.82 | 88 | 64.71 | 73 | 53.68 | 95 | 69.85 | 133 | 97.79 |
|  |  | Male | 31 | 18.45 | 1 | 3.23 | 20 | 64.52 | 26 | 83.87 | 18 | 58.06 | 9 | 29.03 | 27 | 87.10 | 10 | 32.26 | 15 | 48.39 | 14 | 45.16 | 18 | 58.06 | 30 | 96.77 |
|  |  | Non-binary | 1 | 0.60 | 0 | 0.00 | 1 | 100.00 | 1 | 100.00 | 1 | 100.00 | 0 | 0.00 | 1 | 100.00 | 0 | 0.00 | 0 | 0.00 | 1 | 100.00 | 1 | 100.00 | 1 | 100.00 |
|  | Marital status | Single | 165 | 98.21 | 19 | 11.52 | 115 | 69.70 | 139 | 84.24 | 124 | 75.15 | 72 | 43.64 | 146 | 88.48 | 88 | 53.33 | 101 | 61.21 | 86 | 52.12 | 111 | 67.27 | 161 | 97.58 |
|  |  | Married/Civil union | 1 | 0.60 | 0 | 0.00 | 1 | 100.00 | 0 | 0.00 | 0 | 0.00 | 0 | 0.00 | 1 | 100.00 | 0 | 0.00 | 0 | 0.00 | 1 | 100.00 | 1 | 100.00 | 1 | 100.00 |
|  |  | Co-habiting | 2 | 1.19 | 0 | 0.00 | 2 | 100.00 | 2 | 100.00 | 2 | 100.00 | 1 | 50.00 | 2 | 100.00 | 2 | 100.00 | 2 | 100.00 | 1 | 50.00 | 2 | 100.00 | 2 | 100.00 |
| Academic progression | Year on study | 1st year | 53 | 31.55 | 11 | 20.75 | 45 | 84.91 | 48 | 90.57 | 42 | 79.25 | 22 | 41.51 | 43 | 81.13 | 29 | 54.72 | 32 | 60.38 | 32 | 60.38 | 36 | 67.92 | 50 | 94.34 |
|  |  | 2nd year | 33 | 19.64 | 3 | 9.09 | 22 | 66.67 | 27 | 81.82 | 26 | 78.79 | 20 | 60.61 | 32 | 96.97 | 17 | 51.52 | 21 | 63.64 | 19 | 57.58 | 27 | 81.82 | 32 | 96.97 |
|  |  | 3rd year | 7 | 4.17 | 1 | 14.29 | 6 | 85.71 | 7 | 100.00 | 7 | 100.00 | 3 | 42.86 | 7 | 100.00 | 5 | 71.43 | 5 | 71.43 | 3 | 42.86 | 6 | 85.71 | 7 | 100.00 |
|  |  | 4th year | 47 | 27.98 | 2 | 4.26 | 30 | 63.83 | 38 | 80.85 | 34 | 72.34 | 19 | 40.43 | 43 | 91.49 | 25 | 53.19 | 28 | 59.57 | 25 | 53.19 | 34 | 72.34 | 47 | 100.00 |
|  |  | 5th year | 28 | 16.67 | 2 | 7.14 | 15 | 53.57 | 21 | 75.00 | 17 | 60.71 | 9 | 32.14 | 24 | 85.71 | 14 | 50.00 | 17 | 60.71 | 9 | 32.14 | 11 | 39.29 | 28 | 100.00 |
| Social | Children | Yes | 2 | 1.19 | 0 | 0.00 | 1 | 50.00 | 2 | 100.00 | 2 | 100.00 | 1 | 50.00 | 2 | 100.00 | 1 | 50.00 | 1 | 50.00 | 1 | 50.00 | 2 | 100.00 | 2 | 100.00 |
|  |  | No | 166 | 98.81 | 19 | 11.45 | 117 | 70.48 | 139 | 83.73 | 124 | 74.70 | 72 | 43.37 | 147 | 88.55 | 89 | 53.61 | 102 | 61.45 | 87 | 52.41 | 112 | 67.47 | 162 | 97.59 |
|  | Work | Yes | 40 | 23.81 | 6 | 15.00 | 27 | 67.50 | 30 | 75.00 | 30 | 75.00 | 10 | 25.00 | 35 | 87.50 | 20 | 50.00 | 20 | 50.00 | 20 | 50.00 | 28 | 70.00 | 39 | 97.50 |
|  |  | No | 128 | 76.19 | 13 | 10.16 | 91 | 71.09 | 111 | 86.72 | 96 | 75.00 | 63 | 49.22 | 114 | 89.06 | 70 | 54.69 | 83 | 64.84 | 68 | 53.13 | 86 | 67.19 | 125 | 97.66 |
|  | Family problems | Yes | 55 | 32.74 | 12 | 21.82 | 46 | 83.64 | 51 | 92.73 | 49 | 89.09 | 21 | 38.18 | 45 | 81.82 | 25 | 45.45 | 27 | 49.09 | 32 | 58.18 | 41 | 74.55 | 52 | 94.55 |
|  |  | No | 113 | 67.26 | 7 | 6.19 | 72 | 63.72 | 90 | 79.65 | 77 | 68.14 | 52 | 46.02 | 104 | 92.04 | 65 | 57.52 | 76 | 67.26 | 56 | 49.56 | 73 | 64.60 | 112 | 99.12 |
|  | Support networks | Yes | 148 | 88.10 | 14 | 9.46 | 99 | 66.89 | 122 | 82.43 | 108 | 72.97 | 68 | 45.95 | 132 | 89.19 | 82 | 55.41 | 95 | 64.19 | 74 | 50.00 | 99 | 66.89 | 146 | 98.65 |
|  |  | No | 20 | 11.90 | 5 | 25.00 | 19 | 95.00 | 19 | 95.00 | 18 | 90.00 | 5 | 25.00 | 17 | 85.00 | 8 | 40.00 | 8 | 40.00 | 14 | 70.00 | 15 | 75.00 | 18 | 90.00 |
|  | Financial difficulties | Yes | 39 | 23.21 | 9 | 23.08 | 33 | 84.62 | 35 | 89.74 | 31 | 79.49 | 12 | 30.77 | 33 | 84.62 | 18 | 46.15 | 20 | 51.28 | 26 | 66.67 | 32 | 82.05 | 37 | 94.87 |
|  |  | No | 129 | 76.79 | 10 | 7.75 | 85 | 65.89 | 106 | 82.17 | 95 | 73.64 | 61 | 47.29 | 116 | 89.92 | 72 | 55.81 | 83 | 64.34 | 62 | 48.06 | 82 | 63.57 | 127 | 98.45 |
| Healthy habits | Time to reflect | Yes | 102 | 60.71 | 7 | 6.86 | 61 | 59.80 | 81 | 79.41 | 67 | 65.69 | 48 | 47.06 | 94 | 92.16 | 60 | 58.82 | 71 | 69.61 | 48 | 47.06 | 60 | 58.82 | 101 | 99.02 |
|  |  | No | 66 | 39.29 | 12 | 18.18 | 57 | 86.36 | 60 | 90.91 | 59 | 89.39 | 25 | 37.88 | 55 | 83.33 | 30 | 45.45 | 32 | 48.48 | 40 | 60.61 | 54 | 81.82 | 63 | 95.45 |
|  | Physical activity | Yes | 77 | 45.83 | 5 | 6.49 | 46 | 59.74 | 63 | 81.82 | 57 | 74.03 | 40 | 51.95 | 73 | 94.81 | 45 | 58.44 | 50 | 64.94 | 30 | 38.96 | 45 | 58.44 | 76 | 98.70 |
|  |  | No | 91 | 54.17 | 14 | 15.38 | 72 | 79.12 | 78 | 85.71 | 69 | 75.82 | 33 | 36.26 | 76 | 83.52 | 45 | 49.45 | 53 | 58.24 | 58 | 63.74 | 69 | 75.82 | 88 | 96.70 |
|  | Recreational activities | Yes | 120 | 71.43 | 12 | 10.00 | 83 | 69.17 | 99 | 82.50 | 90 | 75.00 | 46 | 38.33 | 107 | 89.17 | 62 | 51.67 | 70 | 58.33 | 61 | 50.83 | 79 | 65.83 | 117 | 97.50 |
|  |  | No | 48 | 28.57 | 7 | 14.58 | 35 | 72.92 | 42 | 87.50 | 36 | 75.00 | 27 | 56.25 | 42 | 87.50 | 28 | 58.33 | 33 | 68.75 | 27 | 56.25 | 35 | 72.92 | 47 | 97.92 |
|  | Alcohol | At least once a week | 23 | 13.69 | 1 | 4.35 | 13 | 56.52 | 19 | 82.61 | 17 | 73.91 | 9 | 39.13 | 21 | 91.30 | 8 | 34.78 | 12 | 52.17 | 11 | 47.83 | 15 | 65.22 | 23 | 100.00 |
|  |  | At least once a month | 61 | 36.31 | 10 | 16.39 | 38 | 62.30 | 48 | 78.69 | 46 | 75.41 | 23 | 37.70 | 57 | 93.44 | 36 | 59.02 | 40 | 65.57 | 30 | 49.18 | 35 | 57.38 | 58 | 95.08 |
|  |  | At least once a year | 23 | 13.69 | 3 | 13.04 | 18 | 78.26 | 19 | 82.61 | 17 | 73.91 | 8 | 34.78 | 17 | 73.91 | 12 | 52.17 | 12 | 52.17 | 15 | 65.22 | 17 | 73.91 | 23 | 100.00 |
|  |  | No | 61 | 36.31 | 5 | 8.20 | 49 | 80.33 | 55 | 90.16 | 46 | 75.41 | 33 | 54.10 | 54 | 88.52 | 34 | 55.74 | 39 | 63.93 | 32 | 52.46 | 47 | 77.05 | 60 | 98.36 |
|  | Tobacco | Yes | 25 | 14.88 | 4 | 16.00 | 17 | 68.00 | 22 | 88.00 | 21 | 84.00 | 7 | 28.00 | 22 | 88.00 | 11 | 44.00 | 13 | 52.00 | 15 | 60.00 | 15 | 60.00 | 24 | 96.00 |
|  |  | No | 143 | 85.12 | 15 | 10.49 | 101 | 70.63 | 119 | 83.22 | 105 | 73.43 | 66 | 46.15 | 127 | 88.81 | 79 | 55.24 | 90 | 62.94 | 73 | 51.05 | 99 | 69.23 | 140 | 97.90 |
|  | Marijuana | Almost every day | 4 | 2.38 | 1 | 25.00 | 3 | 75.00 | 3 | 75.00 | 3 | 75.00 | 0 | 0.00 | 3 | 75.00 | 1 | 25.00 | 2 | 50.00 | 2 | 50.00 | 2 | 50.00 | 3 | 75.00 |
|  |  | At least once a week | 13 | 7.74 | 2 | 15.38 | 10 | 76.92 | 13 | 100.00 | 12 | 92.31 | 5 | 38.46 | 11 | 84.62 | 4 | 30.77 | 7 | 53.85 | 12 | 92.31 | 10 | 76.92 | 13 | 100.00 |
|  |  | At least once a month | 17 | 10.12 | 0 | 0.00 | 9 | 52.94 | 14 | 82.35 | 11 | 64.71 | 6 | 35.29 | 16 | 94.12 | 10 | 58.82 | 11 | 64.71 | 6 | 35.29 | 10 | 58.82 | 17 | 100.00 |
|  |  | At least once a year | 15 | 8.93 | 4 | 26.67 | 9 | 60.00 | 11 | 73.33 | 11 | 73.33 | 4 | 26.67 | 15 | 100.00 | 9 | 60.00 | 11 | 73.33 | 8 | 53.33 | 9 | 60.00 | 14 | 93.33 |
|  |  | No | 119 | 70.83 | 12 | 10.08 | 87 | 73.11 | 100 | 84.03 | 89 | 74.79 | 58 | 48.74 | 104 | 87.39 | 66 | 55.46 | 72 | 60.50 | 60 | 50.42 | 83 | 69.75 | 117 | 98.32 |
|  | Hours on sleep | Less than 3 hours | 3 | 1.79 | 0 | 0.00 | 3 | 100.00 | 3 | 100.00 | 3 | 100.00 | 2 | 66.67 | 3 | 100.00 | 2 | 66.67 | 3 | 100.00 | 1 | 33.33 | 3 | 100.00 | 3 | 100.00 |
|  |  | 3–5 hours | 64 | 38.10 | 13 | 20.31 | 51 | 79.69 | 60 | 93.75 | 55 | 85.94 | 29 | 45.31 | 54 | 84.38 | 37 | 57.81 | 35 | 54.69 | 44 | 68.75 | 53 | 82.81 | 61 | 95.31 |
|  |  | 6–8 hours | 98 | 58.33 | 6 | 6.12 | 62 | 63.27 | 75 | 76.53 | 65 | 66.33 | 41 | 41.84 | 89 | 90.82 | 49 | 50.00 | 63 | 64.29 | 41 | 41.84 | 56 | 57.14 | 97 | 98.98 |
|  |  | Over 8 hours | 3 | 1.79 | 0 | 0.00 | 2 | 66.67 | 3 | 100.00 | 3 | 100.00 | 1 | 33.33 | 3 | 100.00 | 2 | 66.67 | 2 | 66.67 | 2 | 66.67 | 2 | 66.67 | 3 | 100.00 |
| Medical history | Underlying conditions | Yes | 54 | 32.14 | 9 | 16.67 | 43 | 79.63 | 51 | 94.44 | 46 | 85.19 | 24 | 44.44 | 46 | 85.19 | 31 | 57.41 | 33 | 61.11 | 36 | 66.67 | 40 | 74.07 | 52 | 96.30 |
|  |  | No | 114 | 67.86 | 10 | 8.77 | 75 | 65.79 | 90 | 78.95 | 80 | 70.18 | 49 | 42.98 | 103 | 90.35 | 59 | 51.75 | 70 | 61.40 | 52 | 45.61 | 74 | 64.91 | 112 | 98.25 |
|  | Mental health | Depression | 13 | 7.74 | 0 | 0.00 | 13 | 100.00 | 13 | 100.00 | 11 | 84.62 | 4 | 30.77 | 10 | 76.92 | 6 | 46.15 | 7 | 53.85 | 9 | 69.23 | 10 | 76.92 | 13 | 100.00 |
|  |  | Depression and anxiety | 3 | 1.79 | 3 | 100.00 | 3 | 100.00 | 3 | 100.00 | 3 | 100.00 | 1 | 33.33 | 2 | 66.67 | 2 | 66.67 | 1 | 33.33 | 3 | 100.00 | 3 | 100.00 | 3 | 100.00 |
|  |  | Severe stress | 1 | 0.60 | 0 | 0.00 | 1 | 100.00 | 1 | 100.00 | 1 | 100.00 | 0 | 0.00 | 1 | 100.00 | 1 | 100.00 | 1 | 100.00 | 0 | 0.00 | 1 | 100.00 | 1 | 100.00 |
|  |  | ADHD | 1 | 0.60 | 1 | 100.00 | 1 | 100.00 | 1 | 100.00 | 1 | 100.00 | 0 | 0.00 | 0 | 0.00 | 0 | 0.00 | 0 | 0.00 | 1 | 100.00 | 1 | 100.00 | 0 | 0.00 |
|  |  | Eating disorder | 1 | 0.60 | 1 | 100.00 | 1 | 100.00 | 1 | 100.00 | 1 | 100.00 | 0 | 0.00 | 1 | 100.00 | 0 | 0.00 | 0 | 0.00 | 1 | 100.00 | 1 | 100.00 | 1 | 100.00 |
|  |  | Personality disorder | 5 | 2.98 | 1 | 20.00 | 3 | 60.00 | 4 | 80.00 | 5 | 100.00 | 2 | 40.00 | 5 | 100.00 | 4 | 80.00 | 3 | 60.00 | 4 | 80.00 | 4 | 80.00 | 5 | 100.00 |
|  |  | Anxiety disorder | 21 | 12.50 | 3 | 14.29 | 16 | 76.19 | 20 | 95.24 | 20 | 95.24 | 11 | 52.38 | 20 | 95.24 | 11 | 52.38 | 13 | 61.90 | 15 | 71.43 | 15 | 71.43 | 20 | 95.24 |
|  |  | No | 123 | 73.21 | 10 | 8.13 | 80 | 65.04 | 98 | 79.67 | 84 | 68.29 | 55 | 44.72 | 110 | 89.43 | 66 | 53.66 | 78 | 63.41 | 55 | 44.72 | 79 | 64.23 | 121 | 98.37 |

*Notes.* Percentages in outcome columns are calculated within each subgroup. Suicide risk: BHS_SUM_2 ≥ 9. DASS-21 symptomatic: mild-or-greater cut-offs (Depression ≥ 5, Anxiety ≥ 4, Stress ≥ 8 on the respective 7-item raw subscales). Operational categorical thresholds used for the engagement/burnout columns: Vigour ≥ 3.60; Dedication ≥ 3.50; Absorption ≥ 3.67; Engagement total ≥ 3.65; Cynicism ≥ 1.60; Emotional exhaustion ≥ 3.33; Personal fulfilment/academic efficacy ≤ 4.00. The former three-page S1 Table has been consolidated into this single worksheet. The age category is corrected to 18–19 years, and all frequencies were recalculated from the final N = 168 dataset.
