# Supplementary material for: Suicide risk factors in nursing students: Depressive symptoms, academic burnout and academic efficacy after the pandemic
Source: PLOS Ment Health. 2026 Nov 10;3(11):e0000734. doi: 10.1371/journal.pmen.0000734 (PMC13750606; doi:10.1371/journal.pmen.0000734)
Supplement: Supplementary file 3 [file pmen.0000734.s004.docx]

**Response to reviewers:**

***Reviewer 1:***

### **1- Reviewer 1´s comment:** This is an interesting manuscript describing a study exploring possible suicide risk factors in trainee nurses. Whilst the sample size and general topic was of interest, I have some concerns about the written quality of the manuscript (in terms of the commentary for readers, and elaborating some key points), the analysis choices, and the level of detail in places. I hope my comments below are constructive in improving the quality of the manuscript.

***Authors’ response*:** Thank you for your careful and constructive review of our manuscript. We appreciate your comments regarding the conceptualization of suicide risk, the nursing-specific context, measurement choices, statistical analysis, and interpretation of the findings. We have completely revised the manuscript to improve conceptual precision, methodological transparency, reporting of score interpretation, and the Discussion. The specific changes made in response to each comment are detailed below.

**2- Reviewer 1´s comment:** I would like to see greater care with the discussion of 'suicide risk' throughout the article. This is a challenging risk to determine due to the dynamic nature of suicidal thoughts, feelings and behaviours, and I am wary of phrases like 'vulnerable to suicide risk' at the start of the Introduction. Please check throughout.

In relation to this, suicide risk could be better discussed and defined in the introduction given the challenges with this as a construct.

***Authors’ response*:** Thank you for this important observation. We agree that suicide risk should not be presented as a stable or deterministic characteristic. We revised the opening of the Introduction to define suicide risk as a complex and dynamic construct that may vary according to psychological symptoms, life events, interpersonal circumstances, and contextual stressors. We also replaced wording suggesting that nursing students are inherently “vulnerable to suicide risk” with wording referring to exposure to factors associated with elevated suicide-related risk. In addition, we clarified throughout the manuscript that the study operationalizes elevated suicide-related risk using a screening indicator rather than a clinical diagnosis of suicidality.

### **3- Reviewer 1´s comment:** It was not immediately clear on first mention that nursing students might be at high risk because of the academic/emotional demands of their training - being picky, I would argue that other students have similar pressures, so it could be clearer how/why nursing students are at a specific risk. Is this something about the caring nature of their role, the amount of patient contact time nurses tend to have (e.g., versus doctors), or something else about the profession or training? I would like to see the Introduction revised to include a clearer discussion of the nursing context, particularly in Chile, and the likely risk factors associated with nursing training in Chile.

***Authors’ response*:** Thank you for this observation. We revised the Introduction to clarify why nursing education may entail additional stressors beyond those experienced by university students in general. The revised text explains that nursing students combine substantial academic demands with progressively intensive clinical training and direct interaction with patients and families, including exposure to pain, severe illness, suffering, and death. We also clarified that these profession-specific experiences do not imply that nursing students are uniformly at greater suicide risk than students from all other disciplines, but that they may contribute additional psychological strain during training. The Chilean context was also strengthened using previous evidence concerning mental health, academic stress, and suicide-related indicators among nursing and health sciences students.

### **4- Reviewer 1´s comment:** Participants/p3 - please also add the % response rate (the number of students who participated and number invited).

***Authors’ response*:** Thank you for this observation. We have added both the number of eligible students invited and the response rate. All 253 eligible nursing students were invited to participate, and 171 completed the survey, corresponding to a response rate of 67.6%.

### **5- Reviewer 1´s comment:** Measures - please clarify which translated versions of the scales were used - the current draft does not make it clear what the host language was. It may be worth considering how well the translated scales operate in the host country.

***Authors’ response*:** Thank you for this observation. We revised the Instruments subsection to clarify that all questionnaires were administered in Spanish. We now identify the Chilean Spanish versions used for the BHS, MBI-SS, and DASS-21, and clarify that the Spanish-language UWES had previously been used in Spanish-speaking and Chilean university populations. We also explicitly state that no translation of the instruments was performed specifically for the present study.

### **6- Reviewer 1´s comment:** BHS - I am a little sceptical of how much the BHS is a measure of 'suicide risk'. Suicide risk is not wholly well defined here and it could be clearer whether the higher scores are capturing a specific factor associated with depression and suicidality, in the form of severe hopelessness.

***Authors’ response*:** Thank you for this important observation. We agree that the Beck Hopelessness Scale primarily assesses hopelessness and does not directly measure suicidal ideation or suicidal behavior. We therefore revised the manuscript to describe the BHS as a 20-item measure of hopelessness and to clarify that, in this study, BHS scores were used as a screening indicator of elevated suicide-related risk rather than as a diagnostic measure of suicidality. Following the Chilean validation study, scores ≥9 were interpreted as indicating moderate-to-severe hopelessness associated with elevated suicide-related risk. We also explicitly state that the BHS does not directly assess suicidal ideation, suicide attempts, self-harm, or future suicidal behavior and should not be interpreted as a comprehensive clinical assessment of suicidality.

### **7- Reviewer 1´s comment:** Measures - it was not particularly clear what some of the measures captured, especially 'healthy habits'. It would be useful to have this section rewritten into complete paragraphs with clearer indications of what these variables/items were capturing.

***Authors’ response*:** Thank you for this observation. We rewrote the description of the bio-sociodemographic questionnaire and clarified the variables assessed. These included demographic and academic characteristics, financial and family difficulties, support networks, physical and recreational activities, sleep duration, substance use, underlying health conditions, and previous mental health diagnoses. We also replaced the potentially misleading expression “healthy habits” with “health-related behaviors” and clarified that these variables were analyzed individually rather than combined into a composite score.

### **8- Reviewer 1´s comment:** The DASS - this isn't the most sensitive measure of depression, anxiety, and stress, often capturing broader psychological distress and likely skewing towards non-clinical forms of these experiences. I was curious why the authors used the BHS, which has a stronger clinical cut-off, whilst choosing the DASS over the BSS/BDI (especially as the latter measures will better capture suicidality).

***Authors’ response*:** Thank you for this observation. We agree that the DASS-21 assesses self-reported symptoms of depression, anxiety, and stress and is neither a diagnostic instrument nor a direct measure of suicidality. The DASS-21 was selected because it permits a brief, simultaneous assessment of these three dimensions of psychological distress and has been validated among Chilean university students. It therefore served a different, complementary purpose to the BHS. We revised the Instruments subsection to explicitly state that the DASS-21 was used to characterize symptom severity at the group level, rather than to establish psychiatric diagnoses or to assess suicidality directly.

### **9- Reviewer 1´s comment: Page 4 - I'm not clear what 'the prevalence of suicide risk' means;** this seems to be poorly phrased. Suicide risk is often thought to be on a spectrum, and likely very dynamic/changeable over time (e.g., depending on life circumstances, life stress, demands placed on the individual). I think the point in this section is that certain percentages of students scored in the risk range based on the BHS?

***Authors’ response*:** Thank you for this observation. We agree that the expression “prevalence of suicide risk” was insufficiently precise because suicide risk is dynamic and multidimensional. We therefore revised the Abstract, Results, and Discussion to report the proportion of participants who met the predefined BHS threshold for elevated suicide-related risk. Specifically, 14.6% of participants scored within the moderate-to-severe hopelessness range and met the BHS cut-off of ≥9. We now describe these findings as screening-based estimates rather than as the prevalence of a stable clinical condition.

### **10- Reviewer 1´s comment:** Were any measures of past suicidal ideation or behaviours (inc. self-harm) measured in this study? If not, this needs to be considered as a limitation given the role of past experiences in the prediction of future suicidality.

***Authors’ response*:** Thank you for raising this important point. Previous suicidal ideation, suicide attempts, and self-harm behaviors were not assessed in this study. We have now explicitly acknowledged this as a limitation because previous suicidal thoughts and behaviors are relevant factors in the assessment of future suicidality. We also clarified that the BHS-based indicator used in this study should be interpreted as a hopelessness-based screening measure rather than a comprehensive assessment of suicidality.

### **11- Reviewer 1´s comment:** Measures/score ranges - a lot of the results are difficult to interpret as the score ranges are not clearly described (e.g., "75.00% of the sample scored ≥ 77.5 points on total academic engagement" - it is not clear what a score above 77.5 means on this measure, for example). Please attend to this throughout the Methods and Results as it was difficult to determine why some of these scores are important.

***Authors’ response*:** Thank you for this observation. We substantially revised the reporting and interpretation of the UWES and MBI-SS scores. In the Methods, we now explain that UWES scores were expressed as mean item scores ranging from 0 to 6, with higher values indicating greater academic engagement. The Results now report the observed means and standard deviations for vigor, dedication, absorption, and total engagement rather than relying on unclear raw-score thresholds. We also clarified the interpretation of MBI-SS scores, indicating that higher emotional exhaustion and cynicism and lower academic efficacy correspond to greater burnout. The corresponding Results and Discussion sections were revised to report and interpret these mean scores directly.

### **12- Reviewer 1´s comment:** Regression predictors entry - it could be clearer why some variables were removed from the models. 'For parsimony' isn't a great rationale given that multiple regression models including non-sig predictors might still contain important information (controlling for potential variance of key factors in the same model).

***Authors’ response*:** Thank you for this important methodological observation. We agree that statistical significance alone should not be used as sufficient justification for removing a predictor. We therefore revised the Data Analysis and Results sections to distinguish the theoretically specified initial regression model from the reduced model subsequently examined. The initial model included the predictors specified a priori according to the study hypotheses, whereas the reduced model was treated as a secondary exploratory model-refinement analysis. We have also clarified that predictor removal should not be interpreted as confirming or rejecting the original hypothesis and that the reduced model should be interpreted as exploratory rather than confirmatory.

### **13- Reviewer 1´s comment:** Discussion - as mentioned elsewhere, this would benefit from some additional information and commentary throughout

### For example: "Suicide risk in nursing students has been associated with emotionally demanding experiences, such as pain, death, and suffering" - my read of this was that nursing students are potentially highly exposed to patients experiencing pain, significant illness and suffering, as well as patients dying... or is the intended meaning in the text something else?

Please reconsider the phrasing in the Discussion to better explain these points.

***Authors’ response*:** Thank you for this clarification. The intended meaning was indeed exposure to patients experiencing pain, severe illness, suffering, and death during clinical training. We revised the Discussion to make this explicit. The revised text now states that nursing students may encounter emotionally demanding patient-care experiences during clinical training and that such exposure may contribute to psychological strain alongside academic demands. We also clarified that students in the earlier years of training generally have less sustained exposure to clinical environments than students in more advanced stages.

### **14- Reviewer 1´s comment:** There is mention towards the end of the Discussion about the potential interactions between students' wellbeing and other factors (e.g., social factors, external pressures...) - which is a reasonable point. I was curious why no interactions were explored in this study given the potential power of a wellbeing x external pressures interaction on BHS scores.

***Authors’ response*:** Thank you for this valuable suggestion. In response to this comment, we further examined whether the relationship between psychological distress and BHS scores might vary according to academic engagement. A significant Depression × Vigor interaction was identified and incorporated into the revised regression model. The Results now report the interaction term along with its coefficient, effect size, VIF, and tolerance. We also added an interpretation in the Discussion indicating that the association between depressive symptoms and BHS scores varied according to students’ level of vigor. Given the cross-sectional design, we explicitly interpret this interaction as hypothesis-generating rather than causal.

***Reviewer 2:***

1- The manuscript investigates suicide risk correlates in 171 Chilean nursing students using DASS-21, UWES, MBI-SS, and BHS instruments. This is an interesting study, but it has several problems across measurement, analysis, and reporting.

***Authors’ response:*** Thank you for your comment. We have redone all data analysis, ensuring compliance with all standard methodological and reporting requirements. Please accept our sincere apologies for all the problems you encountered while reading the research. All analyses in the revised version of the manuscript were conducted using a 168-participant database, after excluding three outliers based on normal distribution standards, i.e., observations outside the -2 to +2 IQR.

2- The MBI-SS depersonalisation subscale has α = 0.55, which is well below the 0.70 minimum for scientific use, yet it is retained in correlational and descriptive analyses and discussed substantively ("53.20% presented high levels of depersonalisation"). It should be clarified.

***Authors’ response:*** Thank you for your comment. We have renamed all MBISS measures, such as Emotional Exhaustion, Cynicism, and Academic Efficacy, as described in the literature. The former depersonalization scale, now Cynicism, has been examined with the new dataset, after which item 15 was found not reliable enough for the dimension. Therefore, we eliminated this item from the dimension and all subsequent analyses, suggesting that its removal would improve the dimension's reliability. The final Cynicism dimension yielded fair reliability indices, which we consider substantially higher than the previous, and mostly appropriate for the present inquiry (α = 0.64; ω = 0.65).

3- Vigour and absorption correlate at r = 0.836 (Table 5), and this is not evidence of three distinct dimensions but of near-total overlap. It should be clarified.

***Authors’ response:*** Thank you for your comment. Indeed, Vigor correlates strongly with Absorption; in the revised dataset, this correlation is *r* = .81. However, we ensured that these variables were not included simultaneously in the multiple regression model, as they would introduce multicollinearity. In our view, this is more of a construct validity issue that is rather far from our control and beyond our consideration in data analysis, as we did in the multiple regression modeling.

4- "Personal fulfilment" is conventionally reverse-scored in burnout indices (low score = worse outcome), yet the manuscript reports it correlating positively with suicide risk (r = 0.591) and describes "low personal fulfilment" as the risk factor in prose while the table and regression appear to use the raw (non-reversed) scale. This inconsistency between narrative and statistical direction needs to be resolved and explicitly stated; as it stands, a reader cannot verify whether higher raw scores denote more or less fulfilment.

***Authors’ response:*** Thank you for your comment. Personal fulfillment has been renamed Academic Efficacy in agreement with the literature; all items have been reversed to characterize students' level of academic efficacy in the manuscript, rather than its absence. With the updated dataset without extreme outliers, the correlation is 0.57, positive, meaning that being academically effective requires personal effort from nursing students, which can lead to hopelessness and eventually to suicide risk. In other words, it drains the subject’s personal resources. Higher scores indicate greater academic efficacy, while lower scores indicate less academic efficacy. This has been explicitly clarified in the manuscript.

5- Predictors were pruned to p < .001 without pre-registration or theoretical justification, then the paper frames the four survivors as confirming and a priori hypothesis. This is circular: you cannot use a data-driven elimination procedure and then claim hypothesis confirmation from what's left. Report the full model alongside the reduced one.

***Authors’ response:*** Thank you for your comment. We have clarified the full analytical procedure in line with our theoretical understanding and have declared that we tested alternative models in accordance with the theory and previous literature.

6- Table 6 lists χ² (df = 170) for what is described as OLS multiple linear regression. Standard OLS output is an F-statistic with df = (k, n−k−1), not a chi-square with n−1 degrees of freedom. This needs correction or explanation; as written, the regression result (*R²* = 0.52) cannot be independently verified from the reported statistics.

***Authors’ response:*** Thank you for your comment. This was an error. The OLS multiple linear regression statistics in Table 6 (F) have been corrected to include the Standard Error and the Beta estimates for each model predictor. The overall coefficient of determination has also changed to 0.55 when adjusted, as indicated in Table 6.

7- Cohen's d (a standardised mean difference) is inapplicable to a regression R²; the appropriate metric is Cohen's f² (f² = R²/(1−R²) ≈ 1.08 here). Using "d = 1.39" is either a labelling error or a computational error; either way, it needs correction.

***Authors’ response:*** Thank you for your comment. We have amended this and included Cohen's f² for the linear regression model, which is 1.10 and has been reported in the manuscript, which is considered a very large effect size (Cohen, 1988). Cohen's f, the partial effect of each predictor, is now also reported in the revised manuscript.

8- Only one VIF (vigour = 1.844) is reported for a four-predictor model where three of the four (vigour, dedication, personal fulfilment) are substantially intercorrelated (Table 5). Full VIF/tolerance for every retained predictor is required.

***Authors’ response:*** Thank you for your comment. All VIF and Tolerance values have been reported for all the predictors in the revised manuscript.

9- The Results and Discussion report dozens of subgroup breakdowns (by year, sex, work status, marijuana/alcohol use, family problems, etc.) with no correction for multiple testing, and several rest on very small cells (n = 9 third-years driving a 55.6% "suicide risk" figure; n = 7 "no social support" driving 42.9%). These are presented with a precision the sample size cannot support and should be either removed or explicitly caveated as descriptive/hypothesis-generating only.

***Authors’ response:*** Thank you for your comment. We have removed all subgroup breakdowns in the results and discussion that were based on small sample proportions.

10- The Discussion uses language ("erode well-being," "become a trigger for," "increase suicide risk") that implies directionality the design cannot support. This should be systematically softened to associational language throughout.

***Authors’ response:*** Thank you for your comment. All causality statements have been attenuated in concordance with the non-causality and cross-sectional nature of the study design.

11- Classroom-administered, non-probability sampling at one university limits generalisability beyond what is acknowledged; the claimed "95% confidence, 5% margin of error" sample-size calculation is misleading when applied to a non-random sample; this statistical justification does not transfer to a convenience sample and should be removed or reframed.

***Authors’ response:*** Thank you for your comment. The generalizability of the findings has also been toned down and reframed in the manuscript in the limitations of the research, in the discussion, explicitly mentioning the convenience sample design.

12- Reference 43 is a malformed citation ("2022.06.10_Informe.de.la.decada.pdf") lacking author, title, or publisher — this suggests inadequate reference management and should be corrected before further review

***Authors’ response:*** Thank you for your comment. This reference has been corrected in the revised manuscript.
