# Supplementary material for: Suicide risk factors in nursing students: Depressive symptoms, academic burnout and academic efficacy after the pandemic
Source: PLOS Ment Health. 2026 Nov 10;3(11):e0000734. doi: 10.1371/journal.pmen.0000734 (PMC13750606; doi:10.1371/journal.pmen.0000734)
Supplement: Peer review history

# Peer review: Decision Letter 0

**DOI:** 10.1371/journal.pmen.0000734.r001

**Authors:** Wenxi Sun

18 Aug 2026

PMEN-D-26-00349

Suicide risk associated factors in nursing students: burnout, engagement, and mental health in a post-pandemic context

PLOS Mental Health

Dear Dr. Pablo Alejandro Pérez-Díaz,

Thank you for submitting your manuscript to PLOS Mental Health. After careful consideration, we feel that it has merit but does not fully meet PLOS Mental Health’s publication criteria as it currently stands. Therefore, we invite you to submit a revised version of the manuscript that addresses the points raised during the review process.

Please submit your revised manuscript by 15Aug2026. If you will need more time than this to complete your revisions, please reply to this message or contact the journal office at mentalhealth@plos.org. When you're ready to submit your revision, log on to https://www.editorialmanager.com/pmen/ and select the 'Submissions Needing Revision' folder to locate your manuscript file.

Please include the following items when submitting your revised manuscript:

- A letter that responds to each point raised by the editor and reviewer(s). You should upload this letter as a separate file labeled 'Response to Reviewers'.
- A marked-up copy of your manuscript that highlights changes made to the original version. You should upload this as a separate file labeled 'Revised Manuscript with Track Changes'.
- An unmarked version of your revised paper without tracked changes. You should upload this as a separate file labeled 'Manuscript'.

Guidelines for resubmitting your figure files are available below the reviewer comments at the end of this letter.

As the corresponding author, your ORCID iD is verified in the submission system and will appear in the published article. PLOS supports the use of ORCID, and we encourage all coauthors to register for an ORCID iD and use it as well. Please encourage your coauthors to verify their ORCID iD within the submission system before final acceptance, as unverified ORCID iDs will not appear in the published article. *Only*  the individual author can complete the verification step; PLOS staff *cannot*  verify ORCID iDs on behalf of authors.

We look forward to receiving your revised manuscript.

Kind regards,

Wenxi Sun, M.D.

Academic Editor

PLOS Mental Health

**Journal Requirements:**

1. Please amend your detailed Financial Disclosure statement. This is published with the article. It must therefore be completed in full sentences and contain the exact wording you wish to be published.

i. Please clarify all sources of financial support for your study. List the grants, grant numbers, and organizations that funded your study, including funding received from your institution. Please note that suppliers of material support, including research materials, should be recognized in the Acknowledgements section rather than in the Financial Disclosure.

ii. State the initials, alongside each funding source, of each author to receive each grant. For example: "This work was supported by the National Institutes of Health (####### to AM; ###### to CJ) and the National Science Foundation (###### to AM)."

iii. State what role the funders took in the study. If the funders had no role in your study, please state: “The funders had no role in study design, data collection and analysis, decision to publish, or preparation of the manuscript.”

iv. If any authors received a salary from any of your funders, please state which authors and which funders.

2. In the online submission form, you indicated that “The dataset generated and analysed during the current study contains sensitive information related to participants’ mental health and suicide risk. To protect participant confidentiality and comply with the ethical conditions under which the data were collected, the dataset is not publicly available. De-identified data may be made available by the corresponding author upon reasonable request and subject to approval by the institutional ethics committee.”.

All PLOS journals now require all data underlying the findings described in their manuscript to be freely available to other researchers, either

1. In a public repository,

2. Within the manuscript itself, or

3. Uploaded as supplementary information.

This policy applies to all data except where public deposition would breach compliance with the protocol approved by your research ethics board. If your data cannot be made publicly available for ethical or legal reasons (e.g., public availability would compromise patient privacy), please explain your reasons by return email and your exemption request will be escalated to the editor for approval. Your exemption request will be handled independently and will not hold up the peer review process, but will need to be resolved should your manuscript be accepted for publication. One of the Editorial team will then be in touch if there are any issues.

3. Please ensure that your Ethics Statement is available in its entirety at the beginning of your Methods section, under a subheading 'Ethics Statement'. It must include:

i) The name(s) of the Institutional Review Board(s) or Ethics Committee(s)

ii) The approval number(s), or a statement that approval was granted by the named board(s)

iii) (for human participants or donors) - A statement that formal consent was obtained (must state whether verbal/written) OR the reason consent was not obtained (e.g., anonymity).

vi) (for child participants) - Please update your Ethics Statement with confirmation that written informed consent was obtained from the parent/guardian of each participant under 18 years of age.

4. We do not publish any copyright or trademark symbols that usually accompany proprietary names, eg (R), (C), or TM  (e.g. next to drug or reagent names). Please remove all instances of trademark/copyright symbols throughout the text, including JASP®.

5. We have noticed that you have uploaded Supporting Information files, but you have not included a list of legends. Please add a full list of legends for your Supporting Information files after the references list.

If the reviewer comments include a recommendation to cite specific previously published works, please review and evaluate these publications to determine whether they are relevant and should be cited. There is no requirement to cite these works unless the editor has indicated otherwise.

**Additional Editor Comments (if provided):**

[Note: HTML markup is below. Please do not edit.]

Reviewers' comments:

Reviewer’s Responses to Questions

**Comments to the Author**

1. Does this manuscript meet PLOS Mental Health’s publication criteria?

Reviewer #1: Partly

Reviewer #2: Yes

**********

2. Has the statistical analysis been performed appropriately and rigorously?

Reviewer #1: N/A

Reviewer #2: No

**********

3. Have the authors made all data underlying the findings in their manuscript fully available (please refer to the Data Availability Statement at the start of the manuscript PDF file)?

The PLOS Data policy

Reviewer #1: No

Reviewer #2: Yes

**********

4. Is the manuscript presented in an intelligible fashion and written in standard English?

Reviewer #1: Yes

Reviewer #2: No

**********

**Reviewer #1:**  This is an interesting manuscript describing a study exploring possible suicide risk factors in trainee nurses. Whilst the sample size and general topic was of interest, I have some concerns about the written quality of the manuscript (in terms of the commentary for readers, and elaborating some key points), the analysis choices, and the level of detail in places. I hope my comments below are constructive in improving the quality of the manuscript.

Comments:

1. I would like to see greater care with the discussion of 'suicide risk' throughout the article. This is a challenging risk to determine due to the dynamic nature of suicidal thoughts, feelings and behaviours, and I am wary of phrases like 'vulnerable to suicide risk' at the start of the Introduction. Please check throughout.

In relation to this, suicide risk could be better discussed and defined in the introduction given the challenges with this as a construct.

2. It was not immediately clear on first mention that nursing students might be at high risk because of the academic/emotional demands of their training - being picky, I would argue that other students have similar pressures, so it could be clearer how/why nursing students are at a specific risk. Is this something about the caring nature of their role, the amount of patient contact time nurses tend to have (e.g., versus doctors), or something else about the profession or training? I would like to see the Introduction revised to include a clearer discussion of the nursing context, particularly in Chile, and the likely risk factors associated with nursing training in Chile.

3. Participants/p3 - please also add the % response rate (the number of students who participated and number invited).

4. Measures - please clarify which translated versions of the scales were used - the current draft does not make it clear what the host language was. It may be worth considering how well the translated scales operate in the host country.

5. BHS - I am a little sceptical of how much the BHS is a measure of 'suicide risk'. Suicide risk is not wholly well defined here and it could be clearer whether the higher scores are capturing a specific factor associated with depression and suicidality, in the form of severe hopelessness.

6. Measures - it was not particularly clear what some of the measures captured, especially 'healthy habits'. It would be useful to have this section rewritten into complete paragraphs with clearer indications of what these variables/items were capturing.

7. The DASS - this isn't the most sensitive measure of depression, anxiety, and stress, often capturing broader psychological distress and likely skewing towards non-clinical forms of these experiences. I was curious why the authors used the BHS, which has a stronger clinical cut-off, whilst choosing the DASS over the BSS/BDI (especially as the latter measures will better capture suicidality).

8. Page 4 - I'm not clear what 'the prevalence of suicide risk' means, this seems to be poorly phrased. Suicide risk is often thought to be on a spectrum, and likely very dynamic/changeable over time (e.g., depending on life circumstances, life stress, demands placed on the individual). I think the point in this section is that certain percentages of students scored in the risk range based on the BHS?

9. Were any measures of past suicidal ideation or behaviours (inc. self-harm) measured in this study? If not, this needs to be considered as a limitation given the role of past experiences in the prediction of future suicidality.

10. Measures/score ranges - a lot of the results are difficult to interpret as the score ranges are not clearly described (e.g., "75.00% of the sample scored ≥ 77.5 points on total academic engagement" - it is not clear what a score above 77.5 means on this measure, for example). Please attend to this throughout the Methods and Results as it was difficult to determine why some of these scores are important.

11. Regression predictors entry - it could be clearer why some variables were removed from the models. 'For parsimony' isn't a great rationale given that multiple regression models including non-sig predictors might still contain important information (controlling for potential variance of key factors in the same model).

12. Discussion - as mentioned elsewhere, this would benefit from some additional information and commentary throughout

For example: "Suicide risk in nursing students has been associated with emotionally demanding experiences, such as pain, death, and suffering" - my read of this was that nursing students are potentially highly exposed to patients experiencing pain, significant illness and suffering, as well as patients dying... or is the intended meaning in the text something else?

Please reconsider the phrasing in the Discussion to better explain these points.

13. There is mention towards the end of the Discussion about the potential interactions between students' wellbeing and other factors (e.g., social factors, external pressures...) - which is a reasonable point. I was curious why no interactions were explored in this study given the potential power of a wellbeing x external pressures interaction on BHS scores.

**Reviewer #2:**  The manuscript investigates suicide risk correlates in 171 Chilean nursing students using DASS-21, UWES, MBI-SS, and BHS instruments. This is an interesting study, but it has several problems across measurement, analysis, and reporting.

1. The MBI-SS depersonalisation subscale has α = 0.55, which is well below the 0.70 minimum for scientific use, yet it is retained in correlational and descriptive analyses and discussed substantively ("53.20% presented high levels of depersonalisation"). It should be clarified.

2. Vigour and absorption correlate at r = 0.836 (Table 5), and this is not evidence of three distinct dimensions but of near-total overlap. It should be clarified.

3. "Personal fulfilment" is conventionally reverse-scored in burnout indices (low score = worse outcome), yet the manuscript reports it correlating positively with suicide risk (r = 0.591) and describes "low personal fulfilment" as the risk factor in prose while the table and regression appear to use the raw (non-reversed) scale. This inconsistency between narrative and statistical direction needs to be resolved and explicitly stated; as it stands, a reader cannot verify whether higher raw scores denote more or less fulfilment.

4. Predictors were pruned to p < .001 without pre-registration or theoretical justification, then the paper frames the four survivors as confirming an a priori hypothesis. This is circular: you cannot use a data-driven elimination procedure and then claim hypothesis confirmation from what’s left. Report the full model alongside the reduced one.

5. Table 6 lists χ² (df = 170) for what is described as OLS multiple linear regression. Standard OLS output is an F-statistic with df = (k, n−k−1), not a chi-square with n−1 degrees of freedom. This needs correction or explanation; as written, the regression result (R² = 0.52) cannot be independently verified from the reported statistics.

6. Cohen’s d (a standardised mean difference) is inapplicable to a regression R²; the appropriate metric is Cohen’s f² (f² = R²/(1−R²) ≈ 1.08 here). Using "d = 1.39" is either a labelling error or a computational error; either way, it needs correction.

7. Only one VIF (vigour = 1.844) is reported for a four-predictor model where three of the four (vigour, dedication, personal fulfilment) are substantially intercorrelated (Table 5). Full VIF/tolerance for every retained predictor is required.

8. The Results and Discussion report dozens of subgroup breakdowns (by year, sex, work status, marijuana/alcohol use, family problems, etc.) with no correction for multiple testing, and several rest on very small cells (n = 9 third-years driving a 55.6% "suicide risk" figure; n = 7 "no social support" driving 42.9%). These are presented with a precision the sample size cannot support and should be either removed or explicitly caveated as descriptive/hypothesis-generating only.

9. The Discussion uses language ("erode well-being," "become a trigger for," "increase suicide risk") that implies directionality the design cannot support. This should be systematically softened to associational language throughout.

10. Classroom-administered, non-probability sampling at one university limits generalisability beyond what is acknowledged; the claimed "95% confidence, 5% margin of error" sample-size calculation is misleading when applied to a non-random sample; this statistical justification does not transfer to a convenience sample and should be removed or reframed.

11. Reference 43 is a malformed citation ("2022.06.10_Informe.de.la.decada.pdf") lacking author, title, or publisher — this suggests inadequate reference management and should be corrected before further review

**********

what does this mean?). If published, this will include your full peer review and any attached files.

**Do you want your identity to be public for this peer review?**  If you choose “no”, your identity will remain anonymous but your review may still be made public.

For information about this choice, including consent withdrawal, please see our Privacy Policy

Reviewer #1: No

Reviewer #2: No

**********

[NOTE: If reviewer comments were submitted as an attachment file, they will be attached to this email and accessible via the submission site. Please log into your account, locate the manuscript record, and check for the action link "View Attachments". If this link does not appear, there are no attachment files.]

**Figure Resubmissions:**

# Author comment: Author response to Decision Letter 1

**DOI:** 10.1371/journal.pmen.0000734.r002

15 Sep 2026

Attachment Submitted filename: Response to reviewers_final.docx

# Peer review: Decision Letter 1

**DOI:** 10.1371/journal.pmen.0000734.r003

**Authors:** Wenxi Sun

24 Sep 2026

Suicide risk factors in nursing students: depressive symptoms, academic burnout and academic efficacy after the pandemic

PMEN-D-26-00349R1

Dear Pablo Alejandro Pérez-Díaz,

We are pleased to inform you that your manuscript 'Suicide risk factors in nursing students: depressive symptoms, academic burnout and academic efficacy after the pandemic' has been provisionally accepted for publication in PLOS Mental Health.

Before your manuscript can be formally accepted you will need to complete some formatting changes, which you will receive in a follow up email. A member of our team will be in touch with a set of requests.

Please note that your manuscript will not be scheduled for publication until you have made the required changes, so a swift response is appreciated.

IMPORTANT: The editorial review process is now complete. PLOS will only permit corrections to spelling, formatting or significant scientific errors from this point onwards. Requests for major changes, or any which affect the scientific understanding of your work, will cause delays to the publication date of your manuscript.

If your institution or institutions have a press office, please notify them about your upcoming paper to help maximize its impact. If they'll be preparing press materials, please inform our press team as soon as possible -- no later than 48 hours after receiving the formal acceptance. Your manuscript will remain under strict press embargo until 2 pm Eastern Time on the date of publication. For more information, please contact mentalhealth@plos.org.

Thank you again for supporting Open Access publishing; we are looking forward to publishing your work in PLOS Mental Health.

Best regards,

Wenxi Sun, M.D.

Academic Editor

PLOS Mental Health

***********************************************************

-

Reviewer Comments (if any, and for reference):

Reviewer’s Responses to Questions

**Comments to the Author**

Reviewer #1: All comments have been addressed

Reviewer #2: (No Response)

**********

publication criteria?

Reviewer #1: Yes

Reviewer #2: (No Response)

**********

3. Has the statistical analysis been performed appropriately and rigorously?

Reviewer #1: Yes

Reviewer #2: (No Response)

**********

4. Have the authors made all data underlying the findings in their manuscript fully available (please refer to the Data Availability Statement at the start of the manuscript PDF file)?

The PLOS Data policy

Reviewer #1: Yes

Reviewer #2: (No Response)

**********

5. Is the manuscript presented in an intelligible fashion and written in standard English?

Reviewer #1: Yes

Reviewer #2: (No Response)

**********

Reviewer #1: Thank you for addressing my comments.

Reviewer #2: The revised version of the manuscript reads good and the authors responded to my recommendations.

**********

what does this mean?). If published, this will include your full peer review and any attached files.

**Do you want your identity to be public for this peer review?**  If you choose “no”, your identity will remain anonymous but your review may still be made public.

For information about this choice, including consent withdrawal, please see our Privacy Policy

Reviewer #1: No

Reviewer #2: No

**********
